# Different functions of two putative Drosophila α_2_δ subunits in the same identified motoneurons

**DOI:** 10.1038/s41598-020-69748-8

**Published:** 2020-08-13

**Authors:** Laurin Heinrich, Stefanie Ryglewski

**Affiliations:** Institute of Developmental Biology and Neurobiology, Johannes Gutenberg University Mainz, Hanns-Dieter Hüsch Weg 15, 55128 Mainz, Germany

**Keywords:** Neuroscience, Cellular neuroscience, Ion channels in the nervous system, Motor control, Neuronal physiology

## Abstract

Voltage gated calcium channels (VGCCs) regulate neuronal excitability and translate activity into calcium dependent signaling. The α_1_ subunit of high voltage activated (HVA) VGCCs associates with α_2_δ accessory subunits, which may affect calcium channel biophysical properties, cell surface expression, localization and transport and are thus important players in calcium-dependent signaling. In vertebrates, the functions of the different combinations of the four α_2_δ and the seven HVA α_1_ subunits are incompletely understood, in particular with respect to partially redundant or separate functions in neurons. This study capitalizes on the relatively simpler situation in the Drosophila genetic model containing two neuronal putative α_2_δ subunits, straightjacket and CG4587, and one Ca_v_1 and Ca_v_2 homolog each, both with well-described functions in different compartments of identified motoneurons. Straightjacket is required for normal Ca_v_1 and Ca_v_2 current amplitudes and correct Ca_v_2 channel function in all neuronal compartments. By contrast, CG4587 does not affect Ca_v_1 or Ca_v_2 current amplitudes or presynaptic function, but is required for correct Ca_v_2 channel allocation to the axonal versus the dendritic domain. We suggest that the two different putative α_2_δ subunits are required in the same neurons to regulate different functions of VGCCs.

## Introduction

α_2_δ-accessory subunits affect multiple aspects of neuronal high voltage activated calcium channel (HVA VGCC) function^1–5^. Consequently, mutations in α_2_δ genes are cause to neurological conditions such as ataxia^6–8^, epilepsy^9–12^, and neuropathic pain^13–15^. However, despite numerous reports on the roles of α_2_δ subunits in HVA channel trafficking^16,17^, surfacing^18,19^, and biophysical properties^20–24^, the specific in vivo functions, that may result from different α_2_δ/α_1_ combinations, remain incompletely understood. In heterologous expression systems, full HVA calcium current amplitudes require co-expression of α_2_δ and β with the pore forming VGCC α_1_ subunit^5,12,21,24–27^, largely independent of which α_2_δ subunit is used. By contrast, mutations of some α_2_δ subunit genes cause brain disease, rendering functional redundancy unlikely a general scenario in vivo. Although some brain parts co-express multiple α_2_δ subunits^28^, differential spatial expression of α_2_δ subunits has also been observed in the vertebrate brain^29–32^. However, it remains incompletely understood which different combinations of α_2_δ and α_1_ subunits mediate which neuronal functions, and which combinatorial codes of different α_2_δ/α_1_/β combinations regulate which subcellular aspects of HVA channel function in vivo.

The seven vertebrate HVA VGCC genes^33^ comprise two families, four Ca_v_1 channels (Ca_v_1.1–Ca_v_1.4) and three Ca_v_2 channels (Ca_v_2.1–Ca_v_2.3). In combination with four genes each for β- and α_2_δ-subunits (reviewed in^12,34^), this totals to > 100 possible combinations of α_1_/β/α_2_δ HVA VGCC complexes. All so far tested α_1_/β/α_2_δ combinations are functional in heterologous expression systems, but it remains unclear how many of these are used in vivo to regulate different neuronal functions. To address this question we employ specific advantages of the Drosophila genetic model, which contains only one gene each homologous to the vertebrate *Ca*_*v*_*1* and *Ca*_*v*_*2* channel families. *Dmca1D* is homologous to the entire *Ca*_*v*_*1* family, and *Dmca1A*, also named *cacophony*, to the *Ca*_*v*_*2* family^35^. Together with one β- and four genes encoding predicted α_2_δ-subunits, this results in 8 possible combinations per VGCC. Moreover, the relative simplicity, experimental accessibility, and available genetic tools make Drosophila a suitable system to study which α_2_δ/α_1_ combinations regulate which aspects of neuronal HVA channel functional diversity in vivo. We restricted the analysis to CG4587 and stj (dα_2_δ_3_,^36^) because high throughput expression data reveals high expression in the CNS (for stj: flybase.org; for stj and stol: bgee.org, and flyatlas.org). stj aids in neuromuscular synapse assembly during embryonic development^36–38^ and is important for neuromuscular^36–39^ and photoreceptor synaptic transmission^36,37^. Importantly, stj stabilizes the Drosophila Ca_v_2 homolog, cacophony, at the neuromuscular pre-synapse^37^. Furthermore, stj plays a role in a Drosophila model for neuropathic pain^40–42^. However, potential roles of stj in other neuronal compartments than the presynapse remain mostly unclear. For CG4587, no functional data have been reported to date. In contrast to stj and CG4587, dα_2_δ_2_ (Ma2d, CG42817) is reported muscle specific^43^, and a predicted fourth dα_2_δ (CG16868) shows homology to a human α_2_δ-like protein, CACHD1, which associates with Ca_v_3 channels^44^ and shows little mRNA expression in the CNS (flyatlas.org).

This study addresses the function of stj and CG4587 in Drosophila motoneurons (MNs) with well-characterized functions of Ca_v_1 and Ca_v_2 channels in dendrites, the axon, and presynapses^45–48^. We find that both putative α_2_δ subunits mediate different functions. stj is required for normal somatodendritic Ca_v_1 and Ca_v_2 current amplitudes, as well as for correct axonal and presynaptic Ca_v_2 channel function. By contrast, CG4587 is not required for presynaptic function or normal HVA current amplitudes but is critical for correct Ca_v_2 channel allocation to the dendritic domain. We suggest that different putative dα_2_δ subunits are required in the same neuron to cooperatively regulate different aspects of HVA channel function and localization.

## Results

*CG4587* and *CG12295* are two predicted α_2_δ subunits in the Drosophila genome. *CG12295* has been named *straightjacket* (*stj*)^37^ and referred to as *dα*_*2*_*δ*_*3*_^36,38,39^. Stj has important roles for synapse development and function, and pan-neural expression of *UAS-stj* rescues embryonic lethality of *stj* mutants^37,38^. By contrast, CG4587 has neither been investigated nor named. Since *CG4587*^*RNAi*^ reduces climbing speed (see below) we named it stolid (stol). Sequence analysis of both stj and stol shows the presence of two double cache domains as well as a von Willebrand factor A (VWA) as in vertebrate α_2_δ subunits. Vertebrate α_2_δ subunits show either a perfect (α_2_δ_1_ and α_2_δ_2_) or an imperfect MIDAS motif within their VWA domains (α_2_δ_3_ and α_2_δ_4_;^12,49^ which is important for protein–protein interaction. Drosophila stj and stol exhibit imperfect MIDAS motifs within their respective VWA domains^49^. Comparison with vertebrate α_2_δ subunit protein sequences reveals between 26 and 35% sequence similarity at query coverage of 46% to 96% (stj) and 38% to 82% (stol), respectively. stj showed highest sequence similarity to mouse and rat α_2_δ_2_. stol showed highest sequence similarity to mouse α_2_δ_3_. However, sequence homology and functional data on both putative α_2_δ subunits, stj and stol, seem insufficient to claim full resemblance of the Drosophila α_2_δ subunits with specific vertebrate α_2_δ subunits. We use the names stj and stol for the two Drosophila α_2_δ subunits throughout. High throughput expression data reveal expression of both transcripts in the Drosophila ventral nerve cord (VNC) and in the brain (bgee.org and own RNAseq data, see below).

**Figure 1:**
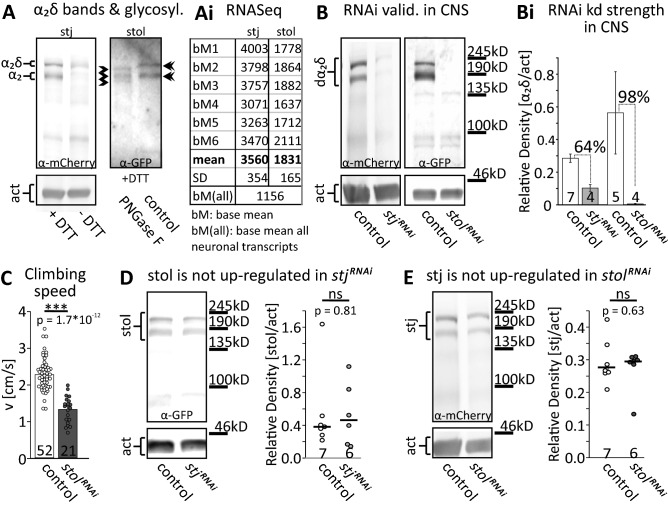
Neuronally expressed stj and stol can effectively be knocked down by RNAi. **(A)** Western blots of endogenous stj^mCherry^ (left) and stol^GFP^ (right) show two bands in the presence of the reducing agent dithiotreitol (DTT) that cleaves disulfide bonds. The tags reside close to the N-terminus on the α_2_ moiety. The upper bands correspond in size to un-cleaved α_2_δ and the lower bands to cleaved α_2_ with mCherry (stj) or GFP-tag (stol), respectively. Without DTT only the larger band at the size of un-cleaved stj^mCherry^ is present **(A,** left blot, right lane). PNGase F de-glycosylation (**A**, right blot, left lane) of purified stol^GFP^ (pulled-down) reduces upper band size by ~ 20 kDa, whereas the lower band splits into two bands, one unchanged and one ~ 20 kDa lower (see arrowheads). **(Ai)** Base mean (bM) expression levels of *stj* (left) and *stolid* (right) mRNA as observed by RNAseq from Drosophila brain with 6 biological replicates each (upper 6 rows). Mean and SD are in rows 7 and 8. Mean expression levels of *stj* and *stol* are higher than the mean of all genes expressed in brain (row 9). **(B, Bi)** Western blotting **(B)** shows effective knock-down of *stj*^*mcherry*^ (left) and of *stol*^*GFP*^ (right) by pan-neural RNAi (*elav*^*c155*^*-GAL4* > *UAS-RNAi; UAS-dcr2*). **(Bi)** Quantification reveals 64% knock-down efficacy for *stj*^*RNAi*^ (control and knock-down, left two bars), and 98% for *stol*^*RNAi*^ (right two bars). Numbers of replicates are indicated in each bar. **(C)** In a negative geotaxis assay, pan-neural *stol*^*RNAi*^ results in a 40% reduction of median climbing speed from 2.3 to 1.4 cm/s as compared to control (N for control: 52, for *stol*^*RNAi*^: 21; ***p < 0.001, Mann–Whitney *U* test). **(D)** Western blot of stol^GFP^ in control (left lane) and with pan-neural *stj*^*RNAi*^ (right lane). Quantification shows that pan-neural *stj*^*RNAi*^ does not affect stol protein level (*p* = 0.81, Mann–Whitney *U* test). **(E)** Western blot of stj^mCherry^ in control (left lane) and with pan-neural *stol*^*RNAi*^ (right lane). Quantification shows that *stol*^*RNAi*^ does not affect stj protein level (*p* = 0.63, Mann–Whitney *U* test). Data in **(D)** and **(E)** are presented as single data points with median.

We tested the in vivo functions of stj and stol on HVA calcium channels by RNAi knock down of the native un-tagged proteins. To assess RNAi knock down efficacy, we performed Western blots. For this, we used animals with endogenously tagged α_2_δ proteins. In controls (genotype: *yw; stj*^*mCherry*^*/ stj*^*mCherry*^; table S1) two bands were detected at the expected sizes of tagged α_2_δ and tagged α_2_ alone (Fig. 1A, left), as also described for vertebrate α_2_δ_1_^2^. It is unlikely that the two bands represent different splice isoforms because the predicted differences in molecular weight of stj and stol isoforms are between 1 and 6 kDa (flybase.org), but the two bands are ~ 25 kDa apart (Fig. 1A, left) which matches the expected molecular weight of the δ moiety of stj (and stol). To provide further support for the interpretation that the two bands are tagged putative α_2_δ and tagged α_2_ alone, we repeated Western blots without the reducing agent dithiotreitol (DTT; see methods) which splits disulfide bonds (Fig. 1A, left). After post-translational cleavage, α_2_δ subunits are re-attached by disulfide bonds between the α_2_ and the δ subunit. Therefore, we predicted only the larger band in the absence of DTT because the δ moiety would not be removed. This was indeed the case (Fig. 1A, left blot, right lane, without DTT). However, vertebrate α_2_δ subunits are heavily N-glycosylated. Different glycosylation states may be an alternative explanation for the appearance of two bands. De-glycosylation of such α_2_δ subunits may account for ~ 25 kDa difference in molecular weight^50^. Therefore, we have conducted Western Blot analysis after enzymatic de-glycosylation experiments with pulled down stol^GFP^ (natively expressed) and subsequent treatment with PNGase F, which is known to cleave N-glycans^50^. We expected only one band in the presence of DTT if the occurrence of two bands was caused by different glycosylation states and two bands if it was caused by cleavage of disulfide bonds, possibly at different molecular weights due to de-glycosylation. PNGase F treatment in the presence of DTT led to the appearance of three bands. The upper one was ~ 17 kDa below the upper band of non-deglycosylated controls. The second band appeared unchanged, while the third one was ~ 20 kDa below the second band of non-deglycosylated controls. The lowest molecular weight band likely represents complete de-glycosylation, whereas the middle band likely represents incomplete de-glycosylation. We conclude that stol is glycosylated, but the presence of two bands after DTT treatment is caused by putative α_2_ and putative α_2_δ. The position of the bands suggests a difference in molecular weight between 17 and 20 kDa upon glycosylation, which is in agreement with glycosylation of stol. stol has 7 putative N-glycosylation sites (according to sequence) and de-glycosylation reduced molecular weight by roughly 20 kDa. In comparison, de-glycosylation of mouse α_2_δ_1_ with PNGaseF leads to a 50 kDa decrease of band height which corresponds to 10 to 14 N-glycosylation sites^51^.

For stj is was shown previously that it is expressed in the Drosophila CNS^36–38^, but for stol this information was not available. We have conducted an RNAseq analysis with Drosophila brain tissue to determine *stj* and *stol* transcript expression levels (Fig. 1Ai). Mean values of normalized counts (= base mean) of 6 biological replicates (bM1-6, Fig. 1Ai, left column) revealed expression of both proteins in the Drosophila brain. Comparison of *stj* and *stol* average base means (bM1-6) to the base mean of all transcripts (bM(all), Fig. 1Ai, bottom) found in Drosophila brain revealed strong expression of both transcripts as both are above bM(all) (stj: 3,560 ± 354 SD; stol: 1,831 ± 165 SD). Average *stol* transcript count was ~ 50% of average *stj* transcript count (Fig. 1Ai). In addition, RNAseq data from FAC sorted larval crawling MNs revealed *stol* and *stj* transcript expression (*stj*: 5,943 ± 1,234 SD; *stol*: 1,490 ± 942 SD; data kindly provided by Dr. JY Roignant, University of Lausanne), further demonstrating that both are expressed in the larval MNs under investigation. To address the functional consequences of stj and stol malfunction in MNs, we targeted *stj* and *stol UAS-RNAi* transgenes specifically to MNs only. Knock down efficacy was estimated by Western blotting following expression of either *stj*^*RNAi*^ or *stol*^*RNAi*^ under the control of the pan-neural driver *elav*^*C155*^*-GAL4* (Fig. 1B,Bi). *UAS-dcr2* was included for enhancement of RNAi knock down efficacy^52^; for flies see below and methods, tables S1 and S2). Although transgene expression levels in MNs may differ from average pan-neural expression levels, this approach yields a reasonable estimate of knock down efficacy. Knock down efficacy was 64% on average for *elav*^*C155*^*-GAL4* > *stj*^*mCherry*^*/UAS-stj*^*RNAi*^*; UAS-dcr2* (Fig. 1Bi, left two bars) and 98% on average for *elav*^*C155*^*-GAL4* > *stol*^*GFP*^*/UAS- stol*^*RNAi*^*; UAS-dcr2* (Fig. 1Bi, right two bars).

For the following reasons stj and stol likely mediate different functions: Stj loss of function is embryonic lethal but an ~ 98% pan-neural RNAi knock down of stol (genotype: *elav*^*C155*^ > *stol*^*RNAi*^*; UAS-dcr2*) is viable, although it significantly reduces the speed of locomotion (Fig. 1C). In addition, *stj*^*RNAi*^ targeted to adult dorsolongitudinal wing depressor muscle (DLM) MNs (genotype: *23H06-GAL4* > *UAS-stj*^*RNAi*^*; UAS-dcr2*) causes inability to fly, but *stol*^*RNAi*^ in the same MNs (genotype: *23H06-GAL4* > *UAS-stj*^*RNAi*^*; UAS-dcr2*) does not abolish flight. Finally, pan-neural RNAi of either putative α_2_δ subunit did not result in compensatory up-regulation of the other one in vivo as revealed by Western Blotting for stj following pan-neural knock down of stol and vice versa (Fig. 1D,E, genotypes: *elav*^*C155*^ > *stol*^*GFP*^*/stj*^*RNAi*^*;UAS-dcr2* and *elav*^*C155*^ > *stj*^*mCherry*^*/stol*^*RNAi*^*;UAS-dcr2*). However, this does not preclude compensatory capacity of one putative α_2_δ subunit for the other if expressed at the right place, time and strength. Nevertheless, we hypothesize that both putative α_2_δ subunits are required in MNs for different functions.

### stj but not stol is required for normal MN Ca_v_1-like and Ca_v_2-like current amplitudes in vivo

**Figure 2:**
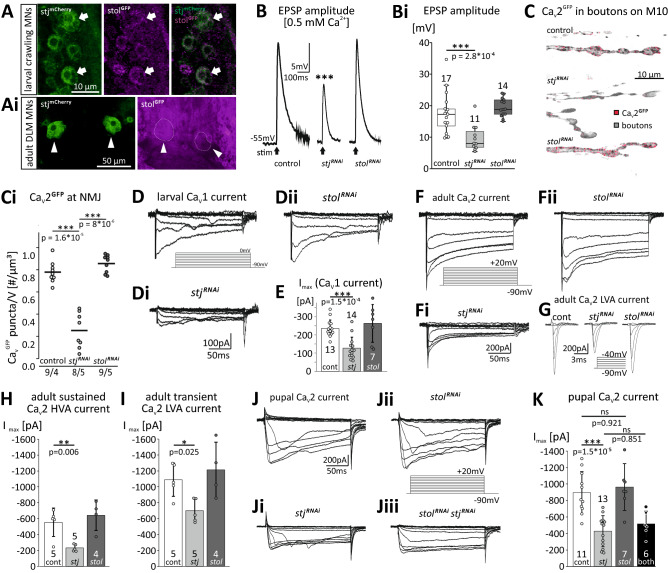
stj but not stol is required for normal MN Ca_v_1- and Ca_v_2-like current amplitudes. Larval **(A)** and adult **(Ai)** Drosophila MN somata show expression of endogenously tagged stol^GFP^ (magenta) and stj^mCherry^ (green). **(B, Bi)** Compared to control (left trace; white box, n = 17) *stj*^*RNAi*^ (middle trace, light gray box, n = 11) but not *stol*^*RNAi*^ (right trace, dark gray box, n = 14) reduces muscle EPSP amplitude in response to MN stimulation (***p < 0.001, Kruskal Wallis ANOVA, Dunn’s post-hoc test). **(C, Ci)** MN axon terminal boutons on larval muscle 10 contain stj^mCherry^ puncta (**C**, top), which are depleted by *stj*^*RNAi*^ (middle), but not by *stol*^*RNAi*^ (bottom). **(Ci)** stj^mCherry^ labeling intensity is significantly reduced by *stj*^*RNAi*^ (light gray, ***p > 0.001) but not by *stol*^*RNAi*^ (dark gray, *p* = 0.21). **(D–E)** Larval MN Ca_v_1 current (**D,** control, 200 ms command voltage steps in 10 mV increments from − 90 to 0 mV) is reduced by *stj*^*RNAi*^**(Di)**, but not by *stol*^*RNAi*^**(Dii)**. Quantification **(E)** reveals 46% amplitude reduction with *stj*^*RNAi*^ (n = 14; ***p = 1.5*10^−4^, one-way ANOVA, LSD post-hoc test) but not by *stol*^*RNAi*^ (n = 7) as compared to control (n = 13). **(F–I)** Adult MN5 HVA and LVA calcium currents mediated by the Drosophila Ca_v_2 homolog cacophony. **(F)** Control HVA current (200 ms steps from − 90 mV to + 20 mV in 10 mV increments) is reduced by *stj*^*RNAi*^**(Fi)**, but not by *stol*^*RNAi*^**(Fii)**. **(G)** Control LVA current (left, steps from − 90 mV to − 40 mV) is reduced by *stj*^*RNAi*^ (middle) but not by *stol*^*RNAi*^ (right). **(H–I)** Quantification shows ~ 60% reduction of HVA current amplitude **(H)** by *stj*^*RNAi*^ (n = 5, **p = 0.006, one-way ANOVA, LSD post-hoc test), but not by *stol*^*RNAi*^ (n = 4) and 36% reduction of LVA current **(I)** by *stj*^*RNAi*^ (*p = 0.025, one-way ANOVA, LSD post-hoc test) but not by *stol*^*RNAi*^ . **(J–K)** Pupal MN5 Ca_v_2 current (**J; K** white bar, n = 11) is 53% smaller following *stj*^*RNAi*^ (**Ji; K** light gray bar, n = 13; ***p = 1.5*10^−5^, one-way ANOVA, Tukey post-hoc test) but unaffected by *stol*^*RNAi*^ (**Jii; K**, dark gray bar, n = 7). Double *stj*^*RNAi*^*stol*^*RNAi*^ did not reduce Ca_v_2 current amplitude further than *stj*^*RNAi*^ alone (**Jiii, K**, black bar, n = 6; *p* = 0.851, one-way ANOVA, Tukey post-hoc test). Bar diagrams in E–K show means ± SD.

Both, stj and stol are expressed in the same larval crawling (Fig. 2A) and pupal/adult wing depressor MNs (Fig. 2Ai) as revealed by antibody label of tagged stj^mCherry^ (shown in green) and stol^GFP^ (shown in magenta) in the VNC (where the MN somata are located). We are aware that tagged proteins may be subject to mis-folding and dysfunction as well as mis-localization. However, we judge it unlikely, that insertion of a tag in a native protein leads to ectopic expression of these proteins. Thus, we conclude that stj and stol are both expressed in larval crawling as well as pupal/adult wing depressor MNs. Expression of stj and stol in the same neurons raises the question whether both proteins have differential and/or redundant functions. To assess the functions of stj and stol in MNs we targeted RNAi transgenes to larval MNs (genotypes: *vGlut*^*OK371*^*-GAL4* > *UAS-stj*^*RNAi*^*/UAS-dcr2* and *vGlut*^*OK371*^*-GAL4* > *UAS-stol*^*RNAi*^*/UAS-dcr2*, respectively. vGlut is short for the vesicular glutamate transporter (please note that Drosophila MNs are glutamatergic) and recorded neuromuscular transmission (Fig. 2B,Bi) and somatodendritic Ca^2+^ currents (Fig. 2D–E). An ~ 64% reduction of stj protein expression in MNs by targeted expression of *stj*^*RNAi*^ reduced larval neuromuscular transmission by ~ 50%, as revealed by current clamp recordings of EPSPs from muscle 10 following extracellular stimulation of the motor nerve (Fig. 2B middle trace, 2Bi light gray box). By contrast, *stol*^*RNAi*^ did not reduce the amplitude of neuromuscular transmission (Fig. 2B, right trace, 2Bi, dark gray box), indicating that stol is not required for normal Ca_v_2 channel function in MN axon terminals. This is in agreement with the presence of stj at the larval NMJ^38^. In support of reduced neuromuscular transmission following *stj*^*RNAi*^ but not *stol*^*RNAi*^, we found reduced label of Ca_v_2^GFP^ channels (endogenously GFP-tagged Drosophila Ca_v_2 (= cacophony) channels ^53^,) in muscle 10 neuromuscular boutons following *stj*^*RNAi*^ (Fig. 2C, control, top; stj, middle, and Ci) but not following *stol*^*RNAi*^ (Fig. 2C, bottom, Ci).

To further test whether different α_2_δ subunits regulate HVA currents selectively in different sub-neuronal compartments, or different HVA channels, or both, we next recorded Ca_v_1 and Ca_v_2 somatodendritic Ca^2+^ current by somatic voltage clamp recordings of identified MNs. Larval MNs express Ca_v_2 like channels in the axon terminal active zones^53^, but somatodendritic Ca^2+^ current is mediated by the Ca_v_1 homolog, Dmca1D^35,48,54,55^. By contrast, adult and pupal DLM MNs use the Ca_v_2 homolog, Dmca1A (cacophony)^35^ for both, axon terminal^56^ and somatodendritic Ca^2+^ current^45,46^. For in situ patch clamp recordings in third instar larvae, *UAS-RNAi* transgenes were targeted to specific crawling MNs in a mosaic fashion to be able to record RNAi MNs and controls in the same animals (genotype: *eve-GAL4.RN2-GAL4/UAS-mCD8::GFP;Act* >  > *GAL4 UAS-FLP* > *UAS-stj*^*RNAi*^*;UAS-dcr2* and *eve-GAL4.RN2-GAL4/UAS-mCD8::GFP;Act* >  > *GAL4 UAS-FLP* > *UAS-stol*^*RNAi*^*;UAS-dcr2*. The presence of *GAL4* and thus expression of *UAS-RNAi* transgenes was reported by expression of *UAS-mCD8::GFP*; for flies see methods, table S2).

Following *stj*^*RNAi*^, MN Ca^2+^ current amplitudes were decreased on average by 46% in larval crawling MNs (HVA Ca^2+^ current, Fig. 2D,Di,E), by 59% (sustained HVA, Fig. 2F,Fi,H) or 36% (transient LVA, Fig. 2G,I) in adult DLM MNs, and by 53% in pupal DLM MNs (HVA Ca^2+^ current, Fig. 2J,Ji,K), respectively. On the contrary, *stol*^*RNAi*^ did not affect somatodendritic Ca^2+^ current amplitudes, neither larval Ca_v_1-like nor adult or pupal Ca_v_2-like current (Fig. 2Dii-K). Moreover, following knock down of both stj and stol in the same pupal DLM MNs Ca^2+^ current amplitudes reflect that of stj knock down MNs (Fig. 2Jiii,K). In summary, *stj*^*RNAi*^ impairs both presynaptic function as well as somatodendritic Ca^2+^ currents but *stol*^*RNAi*^ does not. Hence, stj seems important for normal Ca^2+^ current amplitudes independent of channel type and developmental stage. We next addressed the role of stol for which no functional data exist up to date in Drosophila.

### stj and stol have opposite effects on functional VGCC expression in the axon

**Figure 3:**
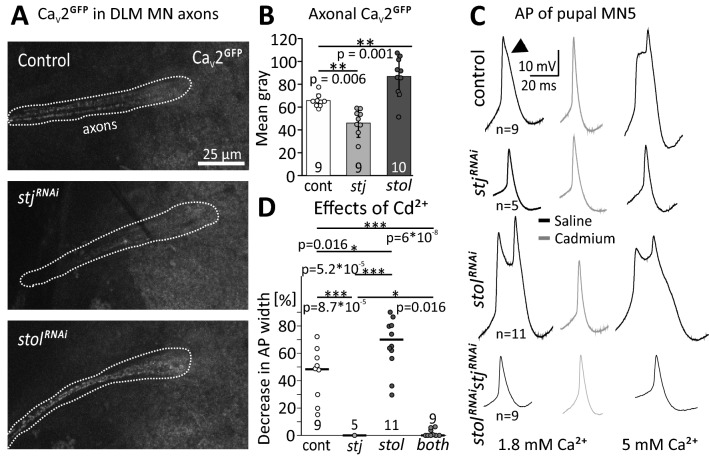
*stj*^*RNAi*^ and *stol*^*RNAi*^ have different effects on axonal Ca_v_2 channel abundance and AP shape. **(A–C)** Projection views (10 optical sections) of DLM MN axons with endogenously tagged Ca_v_2^GFP^ channels, with reported normal function^53^. (**A**) As compared to control (top panel) *stj*^*RNAi*^ (middle panel) decreases Ca_v_2^GFP^ label in DLM MN axons (encircled by dotted white line), whereas *stol*^*RNAi*^ (bottom panel) increases labeling intensity. (**B**) Quantification of mean gray of Ca_v_2^GFP^ puncta in confocal sections reveals a ~ 20% significant decrease in *stj*^*RNAi*^ (light gray bar, **p = 0.006, n = 9) but a ~ 20% significant increase in *stol*^*RNAi*^ (dark gray bar, **p = 0.001, n = 10) as compared to control (white bar, N = 9). Error bars represent SD; statistics, one-way ANOVA, LSD post-hoc test. (**C**) Pupal MN5 action potentials (APs) were recorded in 1.8 (left) and 5 mM Ca^2+^ saline (right) and elicited by somatic square pulse current injection. APs showed a Ca^2+^ shoulder (**C**, top, left trace, see arrow) that was abolished by the VGCC blocker Cd^2+^ (500 µM; **C**, top, gray trace) and broadened in high Ca^2+^**(C**, top, right trace). APs were smaller and narrower in *stj*^*RNAi*^ (**C**, second row, left trace) as compared to control (**C**, top, left trace) and were neither narrowed by Cd^2+^ (**C,** second row, gray trace) nor broadened in 5 mM Ca^2+^ (**C**, second row, right trace). *stol*^*RNAi*^ often caused a double peak (**C**, third row, left trace) and APs were broadened in high Ca^2+^ (**C**, third row, right trace), but abolished in Cd^2+^ (**C**, third row, gray trace). *stj*^*RNAi*^*stol*^*RNAi*^ resulted in APs that were as small as with *stj*^*RNAi*^ but slightly broader, both in 1.8 (**C**, bottom, left trace) and in 5 mM Ca^2+^ (right trace). Cd^2+^ caused a small narrowing effect (**C**, bottom, gray trace). **(D)** Cadmium reduced AP half width significantly more in control (*p = 0.012) than in *stj*^*RNAi*^ (***p < 0.001) but significantly less than with *stol*^*RNAi*^ (*p = 0.016). In *stj*^*RNAi*^*stol*^*RNAi*^ the Cd^2+^ effect was small, but significantly larger as compared to *stj*^*RNAi*^ (*p = 0.016). Diagrams show single data points and medians (Kruskal–Wallis ANOVA, Dunn’s post-hoc test).

**Figure 4:**
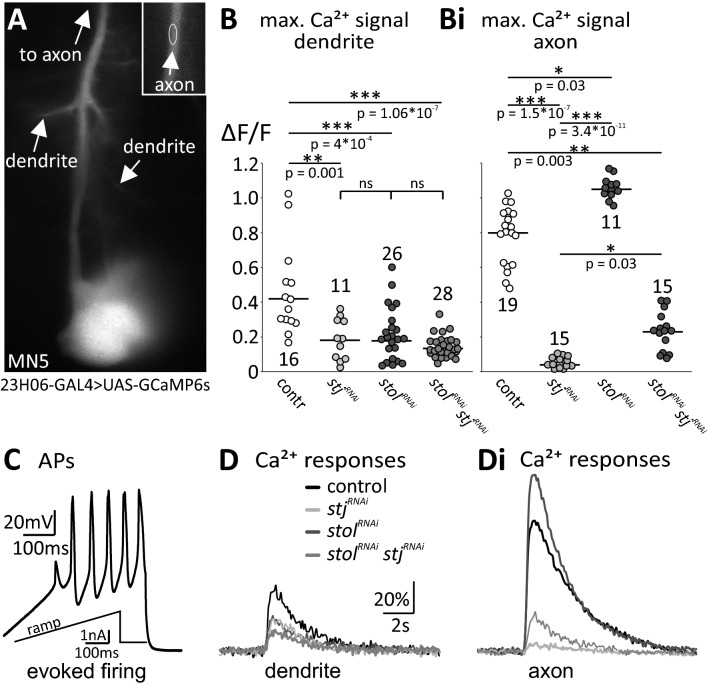
Activity-dependent calcium signals in dendrites are reduced by *stol*^*RNAi*^ and *stj*^*RNAi*^, whereas axonal ones are reduced by *stj*^*RNAi*^ but increases by *stol*^*RNAi*^. (**A**) Pupal MN5 with targeted expression of GCaMP6s. Regions of interest in dendrites and in the axon (inset, upper right corner) are indicated by arrows and white circles. **(B–Di)** Changes in GCaMP6s fluorescence (ΔF/F) in dendrites **(B, D)** and in the axon **(Bi, Di)** upon induced firing by somatic ramp current injection as shown in **(C)**. **(B)** As compared to control (white circles, n = 16) dendritic Ca^2+^ signals are significantly reduced by *stj*^*RNAi*^ (light gray circles; **p = 0.001, n = 11)*, stol*^*RNAi*^ (dark gray circles; ***p < 0.001, n = 26), and *stj*^*RNAi*^*stol*^*RNAi*^ (medium gray circles; ***p < 0.001, n = 28), with no significant differences between both single- and the double knock-down. **(Bi)** By contrast, axonal Ca^2+^ signals are significantly reduced by *stj*^*RNAi*^ (light gray circles; ***p < 0.001, n = 15) but significantly increased following *stol*^*RNAi*^ (**p = 0.001, N = 11) as compared to control (**Bi**, N = 19). *stj*^*RNAi*^*stol*^*RNAi*^ (medium gray circles, N = 15) also significantly reduces axonal Ca^2+^ signals, but significantly less than single *stj*^*RNAi*^ (*p = 0.03). Data in (**B** and **Bi)** are presented as single data points and median. Statistical significance was tested by Kruskal–Wallis ANOVA with Dunn’s post-hoc test. (**D–Di**) Representative traces of (ΔF/F) over time in dendrites **(D)** and in the axon **(Di)** for control (black), *stj*^*RNAi*^ (light gray), *stol*^*RNAi*^ (dark gray), and *stj*^*RNAi*^*stol*^*RNAi*^ (medium gray).

In addition to the prominent role of HVA VGCCs at the presynapse for action potential (AP) triggered synaptic vesicle release and known dendritic functions, axonal functions of HVA channels have been described in both, larval Drosophila MNs^48^ and developing adult Drosophila wing MNs^46^. To visualize axonal Ca_v_2 channels on the level of confocal microscopy, we used Ca_v_2^GFP^ (see also Fig. 2C), which have been reported to function and localize not significantly differently from native channels^53^. The arrangement of all 5 DLM wing MN axons into one axon bundle exiting the VNC towards the DLM wing depressor muscle allows visualization of GFP-tagged Ca_v_2 channels in MN axons by confocal microscopy (Fig. 3A). Axonal Ca_v_2^GFP^ (cac^GFP^) channel label was visibly (Fig. 3A, middle panel) and statistically significantly decreased by targeted *stj*^*RNAi*^ knock down (Fig. 3B). By contrast, *stol*^*RNAi*^ caused increased Ca_v_2^GFP^ channel label in MN axons (Fig. 3A, bottom panel, 3B). Therefore, stj and stol have opposite effects on axonal cacophony channel abundance in DLM MNs. To test whether this was caused by altered transport of cacophony channels, or by functional channels in the membrane we next recorded AP shape in current clamp mode. The DLM MN AP is mainly carried by Na^+^, but it also contains a Ca^2+^-component during specific stages of pupal life^46^. This Ca^2+^ component can be uncovered by bath application of the potent, ubiquitous, and irreversible VGCC blocker Cd^2+^ (500 µM) that reduces AP width in controls (Fig. 3C, upper panel, left two traces, arrow head). Following *stj*^*RNAi*^ AP width was smaller than in controls (Fig. 3C), and bath application of Cd^2+^ did not decrease AP width (Fig. 3C,D), indicating that the Ca^2+^ component was missing (N = 11). These data are in agreement with a reduced expression of functional Ca^2+^ channels in MNs axons following *stj*^*RNAi*^. By contrast, the Ca^2+^ component was even more pronounced, and the AP was broadened following *stol*^*RNAi*^ (Fig. 3C). AP shape was affected to an extent that the Ca^2+^ shoulder (Fig. 3C, top left trace, arrow head^46^) amounted to a double peak that was abolished by application of Cd^2+^ (Fig. 3C, top and third traces, D; decrease in AP width by Cd^2+^). Therefore, together with increased axonal GFP label (Fig. 3A, bottom), *stol*^*RNAi*^ likely increases the density of functional Ca_v_2 channels in MN axons. There are two possible interpretations for these data. (1) increased AP width and activity dependent axonal calcium influx upon *stol*^*RNAi*^ could be caused by a compensatory up-regulation of stj function, although we found that neither stol nor stj expression levels were increased on the CNS-wide level when the other was knocked down (Fig. 1D,E). (2) *stol*^*RNAi*^ may cause less HVA channel trafficking to dendrites, so that more channels are available for the axon. To distinguish these possibilities, we tested double RNAi of *stj* and *stol* (*stj*^*RNAi*^*stol*^*RNAi*^). In *stj*^*RNAi*^*stol*^*RNAi*^, MN APs were broader as compared to *stj*^*RNAi*^ alone, so that Cd^2+^ application resulted in a significantly larger decrease in AP width (Fig. 3C,D). Therefore, stj function could indeed be up-regulated in a compensatory manner in *stol*^*RNAi*^, so that *stj*^*RNAi*^ has a smaller effect. But this interpretation is not consistent with identical effects of stj single- and stj-stol double knock-down on dendritic Ca^2+^ signals (see below, Fig. 4B,D), unless compensatory up-regulation of stj function in response to *stol*^*RNAi*^ occurred only in axons but not in dendrites. Therefore, we consider the second possibility (2) more likely (see discussion).

**Figure 5:**
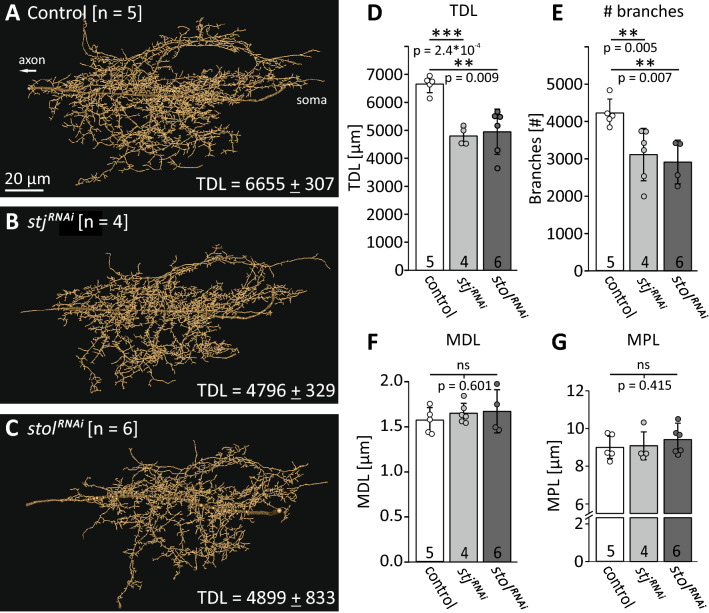
stj and stol affect dendrite development. **(A**–**C)** Reconstructions of adult MN5 dendrite in control (**A**, n = 5) and following *stj*^*RNAi*^ (**B**, n = 4) and *stol*^*RNAi*^ (**C**, n = 6). **(D**–**G)** Morphometric parameters were analyzed. Following *stj*^*RNAi*^ as well as *stol*^*RNAi*^ total dendritic length (**D**, TDL, control: 6,655 ± 307 µm vs. *stj*^*RNAi*^: 4,796 ± 329 µm, ***p = 2.37*10^−4^ and control vs. *stol*^*RNAi*^ 4,899 ± 833 µm, **p = 0.009; one-way ANOVA with Games–Howell post hoc test) as well as the number of dendritic branches (**E**, # branches, control: 4,241 ± 375 µm vs. *stj*^*RNAi*^: 2,914 ± 586 µm, **p = 0.005 and control vs. *stol*^*RNAi*^: 3,122 ± 700 µm, **p = 0.007, one-way ANOVA with LSD post hoc test) are significantly reduced. Other parameters like mean dendrite length (**F**, MDL, control: 1.57 ± 0.14 µm vs. *stj*^*RNAi*^: 1.7 ± 0.2 µm, and control vs. *stol*^*RNAi*^: 1.67 ± 0.11 µm; one-way ANOVA, *p* = 0.601) and mean path length (**G**, MPL, control: 9.02 ± 0.6 µm vs. *stj*^*RNAi*^: 9.09 ± 0.87 µm, and control vs. *stol*^*RNAi*^: 9.41 ± 0.74 µm; Kruskal Wallis ANOVA, *p* = 0.415) are not affected.

A role of α_2_δ subunits on axonal Ca^2+^ influx and thus AP shape is further supported by AP recordings in high Ca^2+^ recording saline (5 mM) which results in even broader APs in controls and following *stol*^*RNAi*^ but not *stj*^*RNAi*^ and hardly in *stj*^*RNAi*^*stol*^*RNAi*^ (Fig. 3C, right traces). Please note that recordings were conducted distant from the spike-initiating zone^57^ from the soma of this unipolar MN, thus reflecting passive propagation along the primary neurite (see Fig. 5 for MN structure). However, our data suggest that stj may be required for normal presynaptic, somatodendritic, and axonal Ca_v_2 channel function, whereas stol may not be required for normal presynaptic Ca_v_2 channel function, but *stol*^*RNAi*^ possibly increases functional axonal Ca_v_2 channel density.

### *stj*^*RNAi*^ reduced dendritic and axonal calcium signals, whereas *stol*^*RNAi*^ reduced dendritic but increased axonal calcium signals

We next tested whether *stj*^*RNAi*^ and *stol*^*RNAi*^ affected also Ca^2+^ signals in dendrites and axons. Somatodendritic Ca^2+^ currents were decreased following *stj*^*RNAi*^ but not following *stol*^*RNAi*^ (Fig. 2), and the Ca^2+^ component in pupal APs was abolished following *stj*^*RNAi*^, but increased following *stol*^*RNAi*^ (Fig. 3). Thus, we expected smaller dendritic and axonal Ca^2+^ influx in *stj*^*RNAi*^ MNs and unaltered dendritic but increased axonal Ca^2+^ influx following *stol*^*RNAi*^. We genetically expressed the Ca^2+^ indicator GCaMP6s^47,58^ in DLM MNs (Fig. 4A, genotype: *23H06-GAL4* > *UAS-IVS-10xUAS-GCaMP6s*) to assess potential changes of activity dependent Ca^2+^ signals by functional imaging. We used pupal MNs because the developing VNC allows better visualization of dendritic processes as compared to the adult one. In these neurons, AP firing causes global Ca^2+^ influx through VGCCs^47^ and was induced by somatic ramp current injection (Fig. 4C). The resulting Ca^2+^ signals were recorded from defined dendritic regions and from the axon (Fig. 4A). Unexpectedly, *stj*^*RNAi*^, *stol*^*RNAi*^*,* and *stj*^*RNAi*^*stol*^*RNAi*^ all reduced dendritic Ca^2+^ signals by about 50% as compared to control but did not differ from each other (Fig. 4B,D, gray circles and traces). This may indicate a potential role of both putative α_2_δ subunits for dendritic VGCC function or membrane localization. However, we cannot exclude effects of stj and stol on other factors that affect activity dependent Ca^2+^ signals. By contrast, reduction in stj and stol expression had opposing effects on axonal Ca^2+^ signals, as predicted based on our results on AP shape (Fig. 4Bi,Di). Axonal Ca^2+^ signal amplitudes were significantly decreased following *stj*^*RNAi*^ (Fig. 4Bi,Di, light gray circles and traces) but significantly increased following *stol*^*RNAi*^ (Fig. 4Bi,Di, dark gray circles and traces). As with AP shape, a possible compensatory action of stj in *stol*^*RNAi*^ cannot be excluded by axonal Ca^2+^ imaging, as following *stj*^*RNAi*^*stol*^*RNAi*^ axonal Ca^2+^ signals are decreased, but remain significantly larger than in single *stj*^*RNAi*^ (Fig. 4Bi,Di, middle gray circles and traces). However, as mentioned above, dendritic Ca^2+^ signals are reduced to the same degree by *stj*^*RNAi*^ and by *stj*^*RNAi*^*stol*^*RNAi*^ (Fig. 4B). This argues against increased stj function upon *stol*^*RNAi*^.

Given that following *stj*^*RNAi*^ Ca^2+^ entry was reduced in all compartments tested, this indicates a principal role for stj to target and/or surface VGCCs to the membrane or to increase channel conductance. Our finding that *stj*^*RNAi*^ reduces the amount of axonal Ca_v_2^GFP^ label (Fig. 3A,B) supports the interpretation that stj is required for channel targeting/and or surfacing. However, we cannot exclude additional roles of stj in increasing channel conductance. By contrast, *stol*^*RNAi*^ specifically reduces dendritic but not axonal Ca^2+^ signals, which suggests a more specific role of stol for targeting VGCCs to the dendritic domain. Reduced Ca_v_2 channel targeting to dendrites in *stj*^*RNAi*^ as well as *stol*^*RNAi*^ is further supported by concomitant dendritic growth defects (Fig. 5), although there are many possible mechanisms that can potentially explain effects on dendrite growth. It has previously been demonstrated that Ca_v_2 channels are required for local^47^ and for global dendritic growth regulation^46,59^ in Drosophila DLM flight MNs. *stj*^*RNAi*^ as well as *stol*^*RNAi*^ phenocopy direct RNAi knock down of Drosophila Ca_v_2 channels in DLM MN5^46^. Similar to RNAi knock down of Ca_v_2 channels in the DLM MN5, both *stj*^*RNAi*^ as well as *stol*^*RNAi*^ cause a significant decrease in total dendritic length (Fig. 5A–E; TDL control: 6,655 ± 307 µm, white bars, *stj*^*RNAi*^: 4,796 ± 329 µm, light gray bars; *stol*^*RNAi*^: 4,899 ± 833 µm, dark gray bars) and in the number of branches (Fig. 5E, # branches, control: 4,241 ± 375 µm, *stj*^*RNAi*^: 2,914 ± 586 µm, *stol*^*RNAi*^: 3,122 ± 700 µm) as revealed by intracellular dye fill and subsequent quantitative dendritic architecture analysis^60^. By contrast, mean dendritic branch length (MDL) and mean path length (MPL) are not affected (Fig. 5F, MDL: control: 1.57 ± 0.14 µm, *stj*^*RNAi*^: 1.7 ± 0.2 µm, *stol*^*RNAi*^: 1.67 ± 0.11 µm and Fig. 5G, MPL: control: 9.02 ± 0.6 µm, *stj*^*RNAi*^: 9.09 ± 0.87 µm, *stol*^*RNAi*^: 9.41 ± 0.74 µm). Therefore, *stj*^*RNAi*^ and *stol*^*RNAi*^ do not seem to affect dendritic territory borders or dendritic branch elongation, but may cause a significant reduction in new dendritic branch formation or maintenance, which may result in reduced total length.

## Discussion

In this study we find that stol and stj mediate different functions in the same identified Drosophila MNs. stj is required for normal MN presynaptic function, somatodendritic Ca_v_1 or Ca_v_2 current amplitudes, and axonal and dendritic Ca^2+^ signals. This is in agreement with a general role of stj in Ca_v_1 and Ca_v_2 channel function in all neuronal compartments. Given that in Drosophila flight MNs, the Ca_v_2 homolog, cacophony, mediates both HVA and LVA calcium currents^45^, *stj*^*RNAi*^ reduces Ca_v_2 HVA and LVA current amplitudes. We have not tested whether *stj*^*RNAi*^ affects also LVA currents mediated by the Drosophila Ca_v_3 homolog, DmαG, because α_2_δ subunits typically do not affect Ca_v_3 currents. The human α_2_δ-like protein CACHD1 interacts with Ca_v_3 LVA channels, and CG16868 was suggested to represent the Drosophila ortholog^44^. It will be interesting to test whether CG16868 interacts with the Drosophila Ca_v_3 homolog, DmαG^45^, but our RNAseq shows little expression of CG16868 in the brain. Stol and stj show far less sequence similarity to CACHD1 (< 30% identity at < 20% query cover), and we did not further investigate it in this study.

*stj* has previously been reported essential for Drosophila neuromuscular synapse development and function. During embryonic development, loss of stj function impairs early steps of synapse formation before calcium channels arrive at the presynaptic terminal, as well as the subsequent recruitment of Ca_v_2-like channels to the active zone^38^. Moreover, at mature larval neuromuscular junctions, stj is required for rapid induction and continuous expression of presynaptic homeostatic potentiation^39^. Our data indicate that stj is not only required for Ca_v_2 channel function at the presynaptic terminal, but also for normal flight MN somatodendritic and axonal Ca^2+^ currents, both of which are mediated by the Drosophila Ca_v_2 channel homolog cacophony^45,46^. Axonal function is in line with findings in mouse sensory neurons, in which knock-out of *α*_*2*_*δ*_*1*_ decreases axonal Ca^2+^ channels^61^. In addition, stj is required for normal larval MN somatodendritic Ca_v_1 current amplitudes^39^, (this study) encoded by the L-type channel homolog *Dmca1D*^48,54,55^. Therefore, stj is required for normal Ca^2+^ current amplitudes in Drosophila MNs independent of sub-neuronal compartment or Ca_v_1 or Ca_v_2 channel type*.* Interactions of the same α_2_δ subunit with Ca_v_1 and Ca_v_2 channels is also reported for vertebrates, albeit in different neurons. In mouse, α_2_δ_2_ assembles with Purkinje neuron P/Q type channels (Ca_v_2.1/α_2_δ_2_/β4 ^25^,) whereas in inner hair cells, it assembles with Ca_v_1.3 channels^4^.

In contrast to stj, stol is not required for normal presynaptic transmission and thus unlikely affects presynaptic Ca_v_2 channel function. Accordingly, *stol*^*RNAi*^ shows no effects on axon terminal Ca_v_2 channel labeling intensity or synaptic transmission. Furthermore, *stol*^*RNAi*^ does not affect somatodendritic Ca_v_1 or Ca_v_2 Ca^2+^ current amplitudes. AP-induced axonal Ca^2+^ signals are nearly eliminated by *stj*^*RNAi*^ but significantly increased by *stol*^*RNAi*^. In fact, *stol*^*RNAi*^ (i) increases the ratio of action potential-induced axonal versus dendritic Ca^2+^ signals in MNs, and (ii) the abundance of Ca_v_2^GFP^ in the axon. Together with our findings that stol does not affect Ca_v_1 and Ca_v_2 current amplitudes, this hints at a function of stol in regulating Ca_v_2 channel trafficking to dendrites, but the underlying mechanisms remain elusive. One possibility is that stol serves linkage to specific motor proteins for transport to the dendritic domain. However, speculations about potential transport or sorting mechanisms reach beyond this study. We speculate that *stol*^*RNAi*^ causes less Ca_v_2 channel trafficking to dendrites, so that at similar overall Ca_v_2 production, more channels are available for the axon. This interpretation is in line with the effects of double *stj*^*RNAi*^*stol*^*RNAi*^, which reduces AP width and axonal Ca^2+^ signals to a lesser degree than *stj*^*RNAi*^ alone. Consequently, *stj*^*RNAi*^, which reduces axonal channel abundance, likely acts on a higher baseline of available channels when stol is reduced.

Note that *stol*^*RNAi*^ increases Ca_v_2 channel abundance along MN axons but not in axon terminals. A possible explanation could be space constraints. Given that Ca_v_2 channels are roughly 10 nm in diameter, additional channels may not fit into the presynaptic active zone. Alternatively, limited availability of interacting presynaptic scaffold proteins in the active zone may not allow additional functional presynaptic Ca_v_2 channels.

In sum, our in vivo analysis indicates that at least in Drosophila MNs different putative α_2_δ subunits are required to regulate different aspects of voltage gated Ca^2+^ channel function and/or localization. At present, it remains unclear whether α_2_δ subunits generally mediate distinct or partially redundant functions in the same neurons^28^. On the one hand, findings from heterologous expression of vertebrate α_2_δ proteins show that multiple different α_2_δ subunits can increase HVA current amplitudes^11^, though different functions of different α_2_δ subunits on current properties have been reported^62,63^. On the other hand, loss-of-function of a specific α_2_δ subunit in brain regions that express multiple α_2_δ subunits often causes mild phenotypes, indicating at least partially redundant functions^28^. However, in vivo, different vertebrate α_2_δ subunits also show differential expression in different brain regions^29–32^, and some neurons express predominantly only one α_2_δ protein^4,28,64^. For example, Ducky mice with α_2_δ_2_ loss-of-function display cerebellar dysfunction, likely due to loss of α_2_δ_2_ interactions with Ca_v_2.1 channels, which cannot be compensated for by other α_2_δ subunits^25,65^. α_2_δ_2_ is also required for normal Ca_v_1.3 currents in inner hair cells (IHCs), whereas knock-out of α_2_δ_3_ seems to have no effect on mature IHC Ca_v_1.3 currents^64^. Accordingly, mutations in some mammalian *α*_*2*_*δ* genes cause brain diseases^6–8^, including epilepsy ^66^, ataxia^67^, allodynia and hyperalgesia^68,69^. Specifically, α_2_δ_1_ was found to be up-regulated following induced peripheral nerve injury in neuropathic pain models^70–72^, and α_2_δ_1_-blocking gabapentinoids reduce neuropathic pain^73,74^. As mentioned above, α_2_δ_2_ subunits are required for normal hearing^4^. This indicates that in vivo, impaired function of at least some α_2_δ subunits is not compensated for by others*.* Similarly, *stj* null mutants are embryonic lethal^36–38^. In addition, *stj*^*RNAi*^ targeted specifically to flight MNs causes inability to fly, and pan-neural *stol*^*RNAi*^ significantly reduces Drosophila climbing speed.

At present, it remains unknown whether specific α_2_δ subunits have the capacity to rescue the loss of another one when expressed at the correct space, time, and strength. Our data on two putative Drosophila α_2_δ subunits that are natively expressed in the same MNs indicate different and non-redundant functions. However, it remains to be tested whether forced overexpression of *UAS-stj* in *stol* mutant MNs under the control of stol regulatory regions can provide rescue of stol function, or vice versa, but this requires the production of new transgenes and fly strains. Nonetheless, in vivo, knock-down of one is not compensated for by the other one, which is in agreement with different brain diseases resulting from specific mutations of single α_2_δ subunits. This underscores the importance for in vivo studies to unravel the combinatorial code by which different α_2_δ/α_1_interactions mediate functional Ca^2+^ channel diversity in different types of neurons and different sub-neuronal compartments.

In general, stj and stol do not necessarily resemble vertebrate α_2_δ subunits in a 1:1 fashion. Although Drosophila stj and stol contain the essential functional domains of vertebrate α_2_δ subunits, like the MIDAS motif, the von Willebrandt factor A (VWA), and the cache domains, sequence homology is not high enough to unambiguously match each vertebrate α_2_δ subunit with a specific Drosophila one. Based on functional analysis so far available, one might speculate that vertebrate α_2_δ_1_ and Drosophila stj are functional pendants, because both are required for Ca_v_2 channel targeting to axon terminals (this study ^5,38^;), both increase calcium channel abundance in the axonal membrane (this study;^61^, both increase Ca_v_2 current amplitude (this study ^18,19^;), both play roles in the development of excitatory synapses independent of calcium channel function^38,75,76^, and both are implicated in models of neuropathic pain and nerve injury^40–42,70–72^.

## Materials and methods

### Animals

*Drosophila melanogaster* were reared at 25 °C, on a 12/12hrs light/dark cycle, in plastic vials on a cornmeal, glucose, yeast, agar diet (for 6 L: 725.69 g glucose, 343.06 g cornmeal, 66 g Agar and 181.38 g active dry yeast; after cooling to 70 °C 76.25 ml Tegosept (10% in 100% ethanol) were added. After cooling to 65 °C 3.5 g ascorbic acid were added). Food was covered and left for 1 day at 4 °C to cool and harden.

Experiments were carried out either in 2–5 day-old adult male and female, pupal stage P8^77^, and third instar larval animals of varying genotypes (for full list of genotypes see tables S1 and S2).

The Drosophila gene *CG4587* is predicted to encode an α_2_δ subunit (Flybase) but until now no functional data existed, and it was not given a name. Based on slower climbing speed in pan-neural *CG4587*^*RNAi*^ animals, we propose to name *CG4587* “*stolid*”, abbreviated “*stol*”.

### Experimental design

Behavioral analysis (Fig. 1) and image analyses (Figs. 2 and 3) were conducted blindly. All other analyses were conducted with the knowledge of experimental groups.

#### Climbing assay

2–4 day old single male or female virgins were put in separate plastic vials 1 day before testing. The climbing behavior was filmed, while a ruler was placed beside the vial as a measure of length. Gently hitting the vial on the ground will induce upwards climbing behavior due to negative geotaxis. Since flies tend to be more inactive during midday, testing was always done between 9:00am–1:00 pm or 3:00–5:00 pm at 25 °C. The climbing speed was analyzed manually with the Avidemux software. Length and duration of climbing attempts were measured to estimate the climbing speed. Mean climbing speed was calculated from three climbing events per fly.

#### Quantification of axonal cac^GFP^ label

For live detection of axonal cacophony^GFP^ (cac; Ca_v_2^GFP^) label, a genomically tagged and endogenously expressed cacophony carrying an N-terminal GFP-tag was used for which no aberrant function was reported^53^.

For quantification of axonal cac^GFP^ label, all preparations were treated exactly the same way. 2–3 day old flies were dissected and instantly fixated with ice-cold 100% ethanol for 10 min. After washing the preparation with PBS for 10 min it was mounted in Vectashield (Vector Laboratories, Lot# X1215) and directly scanned with a Leica TSC SP8 Laser Scanning Microscope (Leica Microsystems Inc., RRID:SSR_004098) with excitation wavelength at 488 nm (Argon laser). All samples were scanned with a 20 × glycerol objective, a zoom factor of 1.8 for further magnification, a z-step size of 0.3 µm and an image resolution of 1,024 × 1,024 pixels. Furthermore, laser and detector settings were always identical and the laser was always warmed up 1 h before images were taken.

Projection views of the axons from stacks of 10 sections were analyzed with Fiji ImageJ 64 V5. To calculate the intensity of the axonal cac^GFP^ label, a section of the axon, shortly after leaving the VNC, was encircled and the total mean gray value was measured. Per fly, the mean axonal cac^GFP^ intensity was calculated from the axons of both sides.

#### Western Blotting and de-glycosylation

For assessment of RNAi efficacy, Western Blots were conducted. L3 larvae were stunned on ice for 5 min and dissected in ice-cold saline. Afterwards the CNS (stol^GFP^: 20; stj^mCherry^: 30) were collected in 70 µl ice-cold 2xSDS sample buffer with dithiotreitol (DTT) as reducing agent to crack disulfide bonds (25 ml 4 × Tris CI/SDS pH 6.8, 20 ml glycerol, 4 g SDS, 0.31 g DTT, 1 mg Bromophenol Blue, add to 100 ml with ddH_2_0), or without DTT to preserve disulfide bonds between the α_2_ and the δ moieties of the α_2_δ proteins (Fig. 1A). Samples were homogenized and boiled at 96 °C for 3 min. Samples were then stored at − 28 °C.

Discontinuous SDS-PAGE in a large Hoeffer gel chamber with 1.5 mm thickness and 15 pockets with 100 µl volume each was done. A 5% (bis-acrylamide) stacking gel (6.8 ml ddH_2_0, 1.7 ml 30% bis-acrylamide, 1.25 ml 4 × Tris/SDS pH 6.8, 100 µl 10% ammonium persulfate, 10 µl TEMED) and an 8% (bis-acrylamide) running gel (18.6 ml ddH_2_0, 10.7 ml 30% bis-acrylamide, 10 ml 4xTris/SDS pH 8.8, 400 µl 10% ammonium persulfate, 16 µl TEMED) was poured and polymerized at 37 °C. Afterwards the pockets were washed with SDS-glycine-Tris electrophoresis buffer (3 g Tris base, 14.4 g glycine, 1 g SDS, add to 200 ml with ddH_2_O). Samples were again boiled at 96 °C and centrifuged at 10,000*g* for 1 min before loading. As a marker 70 µl of Color Protein Standard Broad Range (New England BioLabs, #P7712S; 25 to 245 kD) diluted 1/7 in SDS sample buffer was loaded. The gel was run at 0.02 A until the dye front passed the stacking gel, then the current was increased to 0.03 A (PowerPac, Bio-Rad).

Proteins were blotted onto nitrocellulose in a large wet tank filled with transfer buffer (18.2 g Tris base, 86.5 g glycine, 900 ml methanol add ddH_2_O to 6L). The blotting of the proteins was done at 4 °C overnight at 40 V (PowerPac, Bio-Rad).

After blotting, the membrane was cut in half at about 80 kDa. The two membrane pieces were washed with ddH_2_O for 10 min, incubated with TBST (10 ml 1 M Tris pH 7.5, 30 ml 5 M NaCl, 1 ml Tween20 add to 1,000 ml with ddH_2_O) 3 times for 20 min and blocked with 10% dried milk-TBST solution or BlockAce-TBST solution (BlockAce, Bio-Rad, #170223) for 2 h. After washing the membrane pieces in TBST for 3 times 20 min, they were incubated separately with primary antibody (245–80 kDa: rabbit anti-GFP, 1:1,000, Thermo Fisher Scientific Cat# A-11122, RRID:AB_221569 / rabbit anti-mCherry, 1:1,000, Abcam, Cat# ab213511, RRID:AB_2814891; 80–25 kDa: mouse anti-actin, 1:10,000, DSHB Cat# jla20, RRID:AB_528068) diluted in 2,5% milk-TBST or 25% BlockAce-TBST solution at 4 °C overnight. Both membrane pieces were then separately washed with TBST 3 times for 20 min before incubation with secondary antibodies (245–80 kDa: goat anti-rabbit IgG, 1:10,000, Jackson ImmunoResearch Labs Cat# 111-035-144, RRID:AB_2307391); 80–25 kDa: goat anti-mouse IgG, 1:4,000, Millipore Cat# 12-349, RRID:AB_390192) diluted in TBST for 2 h at 25 °C. After washing the membrane pieces 3 times for 20 min with TBST and 20 min with TBS membrane was incubated in Immobilon Western Chemiluminescent HRP substrate (Millipore, Cat# WBKLS0500) for 5 min. Bands were detected with a Fusion SL Camera and Fusion software (Vilber Lourmat). For analysis a profile blot of the western was done with Fiji ImageJ V5 and the integrated areas of the bands of interest were measured. The relative densities were calculated by dividing the bands of interest with their respective loading control (actin).

For assessment of glycosylation of stol, Western blots were conducted after pull down of stol^GFP^ protein from larval CNS and subsequent PNGase F treatment. Western blot procedure was as above with the following changes: SDS-PAGE was conducted in mini gel chambers (Biozym, Germany). Per lane 80 larval CNS were collected in ice cold RIPA lysis buffer (10 mM Tris/Cl, 1 mM CaCl_2_, 0.5% NP-40, 0.5% deoxycholic acid, 150 mM NaCl, 10 mM NaF, 20 mM β-glycerophosphate—recipe from Chromotek) with freshly added protease inhibitor cocktail (Roche Diagnostics, Germany) and homogenized manually with a sterile micro pestle on ice. Samples were left for 30 min on ice, then centrifuged at 10,000*g* for 8 min. Supernatant was transferred to fresh reaction tubes and kept at − 28 °C until use. Samples were thawed on ice, combined, and total protein content was determined by BCA assay. After redistribution, protein content was ~ 860 µg per sample for pull down. Samples were topped with 0.5 ml wash buffer (10 mM Tris/Cl pH 7.5, 150 mM NaCl, 0.5 mM EDTA) and added to 20 µl of α-GFP coated magnetic agarose beads (Chromotek, GFP-Trap_MA). Samples were incubated for 2 h at 4 °C on an overhead rocker. Then beads with now attached stol^GFP^ were washed 3 × with wash buffer (s.a.), in a magnetic holder, and then centrifuged carefully at 2,000* g* for 30 s. Wash buffer was then discarded and stol^GFP^ beads were topped with 9 µl ddH_2_O and 1 µl 10 × glycoprotein denaturing buffer (New England Biolabs, NEB), final concentration: 0.5% SDS, 40 mM DTT). Samples were then incubated for 10 min. at 100 °C followed by a short centrifugation step (run up to 10,000 g then let run down again). Supernatant was transferred (tubes in magnetic holder) to fresh pre-chilled tubes, then samples were kept on ice. The following de-glycosylation procedure with PNGase F was conducted according to manufacturer’s instructions (NEB). Control was topped with 6 µl ddH_2_O, 2 µl 10% NP-40, and 2 µl glycobuffer 2 (NEB) without PNGase F. For the test samples 1 µl PNGase F was used plus 5 µl ddH_2_O, 2 µl 10% NP-40, 2 µl glycobuffer 2. Samples were incubated at 37 °C for 2 h. Afterwards, samples were spun down quickly, 5 µl sample buffer was added, and samples were loaded directly (22 µl sample per lane). Gel (5% stacking, 8% running gel) ran at 100 V in electrophoresis buffer (s.a.) until the 45 kDa protein standard (Roti Mark Tricolor, Roth Chemicals, Germany, 10 µl per lane) ran out. Wet transfer onto nitrocellulose membrane was done overnight at 4 °C at 30 V as with normal Western Blot (s.a.). Further treatment was done as described above for stol^GFP^ protein. A loading control was not used, as this was a purified protein.

#### Generation of stj^mCherry^ flies

For Western Blot analysis of RNAi efficacy flies expressing endogenously tagged α_2_δ^78^ were used due to lack of specific antibodies for Drosophila α_2_δ subunits. Rabbit α-GFP and rat α-mCherry antibodies were used (see above). Flies expressing endogenously GFP-tagged stol are commercially available (RRID:BDSC_59289; the GFP-tag resides between amino acids 38 and 39 also in proximity to the N-terminus.). In the stj protein, the mCherry tag is situated close to the N-terminus between amino acids 66 and 67.

Flies with endogenously mCherry-tagged stj were generated using a *Minos* mediated integration cassette (MiMIC)^69^. A MiMIC is flanked by two inverted ϕC31 bacteriophage attP sites and contains a gene-trap cassette and the yellow^+^ marker. ϕC31 expression was driven by the *vasa* promoter. Flies with a MiMIC construct in a coding intron of stj were obtained from Bloomington Drosophila Stock Center (RRID:BDSC_34109). A for the splicing phase (phase 0) compatible plasmid containing the *mCherry* sequence was obtained from the Drosophila Genomics Resource Center (DGRC #1299_*pBS-KA-attB1-2-PT-SA-SD-0-mCherry*;^79^).

Female virgins of a *vasa* integrase line (RRID:BDSC_36312) were crossed with the stj-MiMIC flies (RRID:BDSC_34109). F1 stage 2 embryos were injected with the DNA solution containing the mCherry plasmid (300–400 ng/µl). The injection electrodes (Science Products, GB100TF-8P) were pulled with a Flaming/Brown micropipette puller (Sutter Instruments Co., Model P-97) and broken individually. Injections were conducted with a Femtojet Injector (Eppendorf, cat# 5253000017) in Voltalef 10 s oil. After injection, embryos were covered with Voltalef 3S oil and kept on 25 °C. Hatched larvae were raised on instant fly food (Schlüter Biologie, Cat# 351.205). Every hatched fly was crossed individually with either female virgins or males of a balancer stock (*y*^*1*^w*; Cyo/Sna^*Sco*^). F1 offspring displaying the yellow phenotype were re-crossed with balancer flies to build a stock. All stocks were checked for correct integration of the *mCherry* construct via PCR.

Primer sequences were obtained according to^69^: Orientation-MiL-F: GCGTAAGCTACCTTAATCTCAAGAAGAG; Orientation-MiL-R: CGCGGCGTAATGTGATTTACTATCATAC; mCherry-Seq-F: ACGGCGAGTTCATCTACAAG; mCherry-Seq-R: TTCAGCCTCTGCTTGATCTC. Four different PCR reactions (2 µl 10 × Thermopol buffer, 0.5 µl 10 mM dNTP’s, 0.5 µl F-primer, 0.5 µl R-primer, 0.1 µl Taq polymerase, 1 µl DNA, 5.4 µl ddH_2_O^RNAse free^) had to be performed for each event. The following primer combinations were used: (1) Orientation-MiL-F / mCherry-Seq-R; (2) Orientation-MiL-F / mCherry-Seq-F; (3) Orientation-MiL-R / mCherry-Seq-R; (4) Orientation-MiL-R / mCherry-Seq-F and a touchdown PCR (Biometra, TGradient, Labexchange) was performed: 1x (94 °C, 600 s); 8x (94 °C, 30 s; 68 °C +  − 1 °C, 30 s; 68 °C 90 s); 32x (94 °C, 30 s; 60 °C, 30 s; 68 °C, 90 s); 1x (68 °C, 600 s). PCR products were loaded on a 0.7% agarose gel with ethidium bromide added directly to the gel and run at 70 V (PowerPac, Bio-Rad) for about 60 min. Correct integration was marked by positive primer reactions for the primer combinations 1 & 4).

#### Repairing the stol^RNAi^ stock (Vienna Drosophila Resource Center; VDRC_108150)

As previously described^80^, during generation of VDRC “KK” RNAi stocks in rare cases the RNAi construct integrated into a second landing site (40D) in addition to the intended 30D landing site. Integration of the construct in both sites can lead to expression of a toxic protein called Tiptop (Tio). Thus, in order to prevent unspecific effects, the used KK stocks needed to be tested via PCR. Primer sequences were used as described^70^ (C_Genomic_F: GCCCACTGTCAGCTCTCAAC; NC_Genomic_F: GCTGGCGAACTGTCAATCAC; pKC26_R: TGTAAAACGACGGCCAGT; pKC43_R: TCGCTCGTTGCAGAATAGTCC). Four primer reactions (2 µl 10 × Thermopol buffer, 0.5 µl 10 mM dNTP’s, 0.5 µl F-primer, 0.5 µl R-primer, 0.1 µl Taq polymerase, 1 µl DNA, 5.4 µl ddH_2_O^RNAse free^) had to be done for each tested line (1. C_Genomic_F / pKC26_R; 2. C_Genomic_F / pKC43_R; 3. NC_Genomic_F / pKC26_R; 4. NC_Genomic_F / pKC43_R) and a touchdown PCR was performed: 1x (95 °C, 120 s); 5x (95 °C, 15 s; 68 °C +  − 1 °C, 15 s; 72 °C 50 s); 29x (95 °C, 15 s; 62 °C, 15 s; 72 °C, 50 s); 1x (72 °C, 120 s). PCR products were loaded a 0.7% agarose gel with ethidiumbromide added directly to the gel and run at 70 V (PowerPac, Bio-Rad) for about 60 min.

Integration of the construct into the 40D landing site resulted in a PCR product of approx. 450 bp (C_Genomic_F / pKC26_R), while an empty site resulted in a PCR product of approx. 1,050 bp (C_Genomic_F / pKC43_R). Integration of the construct into the 30D site resulted in a PCR product of approx. 600 bp (NC_Genomic_F / pKC26_R), while an empty site resulted in a PCR product of approx. 1,200 bp (C_Genomic_F / pKC43_R).

For the *stol*^*RNAi*^ stock (FlyBase Cat# FBst0479962, RRID:FlyBase_FBst0479962) the pKC26 vector indeed integrated into both the 30D and 40D site. The unwanted 40D insertion was removed via miotic recombination. Female *stol*^*RNAi*^ virgins (VDRC_108150) were crossed to males of the KK landing line in which both sites were empty (VDRC_60100). Female virgins of the F1 progeny were then crossed to a second chromosome balancer stock. Putatively recombinant offspring could be pre-selected via eye color (red eyes) and were tested for one-sided recombination via PCR as described above.

#### In situ electrophysiology and calcium imaging experiments

Voltage clamp and current clamp experiments^45,46,48,55^ and calcium imaging experiments were carried out as published^47^.

An upright Zeiss Axio Examiner A1 epi-fluoresecence microscope with a 40 × water immersion lens (Zeiss, Germany) with a fixed stage (Narishige) was used. Recordings were done at room temperature (24 °C). Electrophysiological experiments were conducted from crawling MN somata in third instar larvae, and wing depressor MN somata (DLM, specifically MN5) from pupae stage P8 (~ 47–50 h after puparium formation, approx. halfway through pupal development (P50%)) and 2–5 day-old adult *Drosophila melanogaster* of each sex. Selection criterion for P8 was orange eyes as visible through the pupal case^77^. All electrophysiological recordings were carried out in patch clamp whole cell configuration with an Axopatch 200B patch clamp amplifier (Molecular Devices), either in voltage clamp or current clamp mode. Data were digitized at a sampling rate of 50 kHz using a Digidata 1440 analog/digital converter (Molecular Devices) and low pass filtered with a 5 kHz Bessel filter. Data were acquired with pClamp 10.7 software (Molecular Devices).

The ganglionic sheath of the VNC was focally digested and debris was carefully loosened and removed from the MN membrane with 1% *Streptomyces griseus* protease type XIV in saline using a broken patch pipette^81^, and then rinsed thoroughly. Recording patch pipettes were pulled with a PC-10 vertical electrode puller (Narishige) from 1.5 mm outer and 1 mm inner diameter patch clamp glass capillaries without filament (WPI, #PG52151-4). Pipette resistance with Ca^2+^ current recording solutions was ~ 3.5 MΩ for pupal and adult MN5, ~ 4 MΩ for larval MNs, in action potential recording solutions ~ 6 MΩ for pupal and adult MN5, and ~ 6.5 MΩ for larval MNs. For solutions see below. Preparations were perfused with fresh saline (~ 0.5 ml/min) throughout the course of the entire experiment.

#### Recording solutions

Intracellular Ca^2+^ current recording solution (in mM): 140 CsCl, 0.5 CaCl, 2 Mg-ATP, 11 EGTA, 20 TEA-Br, 0.5 4-AP, 10 HEPES; pH was adjusted to 7.24 with 1 N CsOH, osmolality was 327 mOsM/kg.

Extracellular Ca^2+^ current recording solution (in mM): 93 NaCl, 5 KCl, 4 MgCl_2_, 1.8 CaCl_2_, 1.8 BaCl_2_, 30 TEA-Cl, 2 4-AP, 5 HEPES, ~ 35 sucrose. pH was adjusted to 7.24 with 1 N NaOH, osmolality was adjusted to 320 mOsM/kg with sucrose if necessary. TTX was added directly to the bath (the perfusion was halted for 5 min) at 10^−7^ M (adults and pupae) or 4*10^−7^ M (larvae) to block fast Na^+^ current. K^+^ channels were blocked with TEA and 4-AP.

Intracellular action potential recording solution (in mM): 140 K-gluconate, 2 Mg-ATP, 2 MgCl_2_, 11 EGTA, 10 HEPES. pH was adjusted to 7.24 with 1 N KOH, osmolality was adjusted to 300 mOsM/kg with glucose if necessary.

Extracellular action potential recording solution (normal saline; in mM): 128 NaCl, 2 KCl, 4 MgCl_2_, 1.8 CaCl_2_, 5 HEPES, ~ 35 sucrose, pH was adjusted to 7.24 with 1 N NaOH, osmolality was adjusted to 290 mOsM/kg with sucrose if necessary.

Intracellular Ca^2+^ imaging solution (in mM): 140 K-gluconate, 2 Mg-ATP, 2 MgCl_2_, 10 phosphocreatine di tris, 0.3 Na_2_GTP, 10 HEPES. EGTA was omitted because of the presence of GCaMP6s. pH was adjusted to 7.24 with 1 N KOH, osmolality was 313 mOsM/kg.

Extracellular action potential recording solution (normal saline; in mM): 115.8 NaCl, 2 KCl, 4 MgCl_2_, 5 CaCl_2_, 5 HEPES, ~ 35 sucrose, pH was adjusted to 7.24 with 1 N NaOH, osmolality was adjusted to 305 mOsM/kg with sucrose if necessary.

### Voltage clamp and current clamp experiments (incl. Ca^2+^ imaging experiments)

#### For voltage and current clamp recordings

offset was nulled manually while approaching the cell, applying gentle positive pressure to the patch pipette to avoid dilution of the tip with extracellular solution. After gigaseal formation, mode was changed to patch configuration (or on-cell), and the cell was clamped to − 30 mV (for Ca^2+^ current recordings) or − 70 mV (for AP recordings), respectively. Fast capacitance artifacts of the recording electrode were zeroed using the C-slow and C-fast dials of the amplifier, lag was 2 µs. Break-in was achieved by short and quick, gentle suction. Configuration was changed to whole cell, and cell capacitance as well as series resistance were compensated for using the whole cell cap and serial resistance dials of the amplifier. Only recordings with series resistances below 10 MΩ were continued. Usually, series resistance was ~ 8 MΩ. Prediction was set to ~ 98%, and compensation was between 40 and 50%. In Ca^2+^ current experiments, the cell was manually clamped to − 70 mV in 20 mV increments, once all parameters were compensated. This was necessary because the Goldman potential with the given solutions was around 0 mV and therefore far away from the intended holding potential of − 70 mV. Clamping the cell to − 70 mV immediately often results in rupture.

#### Ca^2+^ current recordings

Ca^2+^ currents were recorded in voltage clamp mode. Currents were evoked by 200 ms voltage steps from − 90 to + 20 mV (adult and pupal MNs) or 0 mV (larval MNs) from a holding potential of − 90 mV in 10 mV increments. Linear leak was calculated from the first three voltage steps and subtracted offline. Adult Ca^2+^ current consists of low (LVA) and high voltage activated (HVA) currents. The fast LVA was isolated by addition of the off-artifact to the on-artifact. LVA can only be observed in isolation between − 70 and − 40 mV. HVA activates around − 30 mV and is also carried by cacophony which makes selective block of one component impossible^45^.

#### AP recordings

After break-in, parameters were adjusted as for Ca^2+^ current recordings (s.a.) to get an idea how healthy the cell is. Then we switched to current clamp mode. Only cells with a membrane potential ≤  − 55 mV were used. Pupal (P8) action potentials^46^ were elicited by depolarizing ramp or square current injection. For Ca^2+^ imaging experiments, a 400 ms 1 nA max. amplitude ramp current injection was performed which reliably elicited a train of action potentials.

#### Ca^2+^ imaging

APs were elicited as described above, and the resulting changes in GCaMP6s^58^ fluorescence were recorded and analyzed. An Orca Flash 4.0 LT CMOS camera (C11440-42U; Hamamatsu Photonics K.K.) with HOKAWO 3.10 software was used for image acquisition. Exposure time was 75 ms. Image series were streamed. Raw data were exported to MS Excel, and ΔF/F was calculated by [F(firing)-F(rest)]/F(rest)^47^. Regions of interest (ROI) were chosen in dendrites and axon.

#### Intracellular muscle recordings from L3 larvae

EPSPs were recorded in HL3.1 saline with 0.5 mM Ca^2+^ (62.5 mM NaCl, 10 mM MgCl_2_, 5 mM KCl, 0.5 mM CaCl_2_, 10 mM NaHCO_3_, 5 mM Trehalose, 5 mM HEPES, 35 mM Sucrose; pH 7.24–7.25, osmolality 300–310 mOsM/kg). Electrodes were pulled from borosilicate glass capillaries (WPI, 1B100F-4) with a Flaming/Brown micropipette puller (Sutter Instruments Co., Model P-97). L3 larvae were dissected and the CNS was removed at the end of the dissection procedure by cutting the nerves as close to the CNS as possible. A sharp electrode (tip resistance 30 MΩ) filled with 3 M KCl was placed close to muscle M10 of a thoracic segment. As reference, a chlorinated silver wire was placed inside the bath solution. Offset and capacitance of the electrode were adjusted manually before the electrode was inserted into the muscle. Signals were amplified with an Axoclamp 2B intracellular amplifier in Bridge mode, digitized with a Digidata 1440 and recorded with pClamp 10.7 software (all Molecular Devices). Only data from muscles with a membrane potential of ≤  − 50 mV were used for analysis. To evoke PSPs the respective nerve was sucked into and stimulated by a suction electrode (Sutter, BF100-50-10; broken individually). As reference, a thin silver wire wrapped around the suction electrode was used. Electrical stimuli with a duration of 0.5 ms and the minimal voltage needed (+ 1 V) for action potential generation were applied via an Isolated Pulse Stimulator (Model 2100, A-M Systems) and amplified by a Differential AC Amplifier (Model 1700, A-M Systems). A stimulus train of 0.5 Hz was applied for 20 s. EPSP amplitudes were analyzed with Clampfit 10.7. Per animal, the mean amplitude of 10 EPSPs was calculated.

#### Intracellular dye fill

Adult MN5 was filled as described previously^47^. Adult flies were dissected, and the ganglionic sheath was enzymatically digested. Then the very tip of a sharp glass microelectrode (borosilicate, outer diameter 1 mm, inner diameter 0.5 mm, with filament, Sutter BF100-50-10) pulled with a Sutter P97 Flaming Brown horizontal electrode puller was filled with a 50/50 mixture of TRITC-Dextran 3000 (Thermo Fisher, Cat# D3307) and Neurobiotin (Vector Laboratories Cat# SP-1120-20, RRID:AB_2336606) in 2 M KAcetate. Then the shaft was filled with 2 M KAcetate leaving an air bubble between the dye-loaded tip and the KAcetate to avoid dye dilution. The electrode was connected to an intracellular amplifier (Axoclamp 2B, Molecular Devices) in Bridge mode; tip resistance was ~ 60 MΩ. After impalement of the MN soma with the sharp electrode, the dye was injected iontophoretically into the cell by application of up to 1 nA positive current. Filling quality was judged visually. After completion, the electrode was removed, and the preparation was fixed with 4% paraformaldehyde in phosphate buffered saline (PBS) for 50 min at room temperature. After fixation the preparation was washed at least 6 × 20 min with PBS, then 6 × 20 min with 0.5% PBS-TritonX 100, both shaking. This was followed by incubation in Streptavidin coupled to Cy3 (Thermo Fisher Scientific, Cat# 434315) at a concentration of 1:750 at 4 °C overnight, shaking. The preparation was then rinsed a few times with PBS, and then washed at least 6 × 30 min with PBS. Then the preparation was subjected to an ascending ethanol series (50, 70, 90, 100% ethanol), 10 min each, and then mounted in methylsalicylate on metal slides with an 8 mm hole with a glass cover slip glued to one side with super glue. Preparations were covered with a glass cover slip, which was sealed with nail polish. Reconstruction-ready images are generated using a Leica TSC SP8 confocal laser scanning microscope with a 40x, 1.25 NA oil lens with a 561 nm DPSS laser. Detection range was between 570 and 600 nm. Z-step size was 0.3 µm, zoom 3.5. Voxel dimensions were 86 × 86 × 290 nm (x, y, z). Dendritic structure was reconstructed from confocal image stacks after export to Amira software (AMIRA 4.1.1, FEI, Hillsboro, Oregon, US) with custom plug-ins^82,83^.

### Statistical testing

Statistical analysis was conducted in SPSS Statistics version 23. The distribution of the data was assessed by Shapiro–Wilk test. Two non-related groups with normal distribution were tested with an unpaired Student’s T-test, whereas two non-normally distributed and non-related groups were compared by Mann–Whitney U test. For normally distributed data with several groups (> 2) the variance homogeneity was tested by Levene test and a one-way ANOVA was performed. For groups with homoscedasticity the LSD post hoc test was performed when 3 groups were compared, and the Tukey post-hoc test with 4 groups, whereas a Games-Howell post hoc test was performed for groups with heteroscedasticity. For non-normally distributed data with several groups (> 2), a Kruskal–Wallis ANOVA was conducted and pairwise testing was done by Dunn’s post hoc test or a Median Test of k samples. F-values for one-way ANOVA are reported as ratio (XX) of the between (SSB) and within (SSW) sum of squares of deviations from the mean in the format F(SSB,SSW) = XX. All results of statistical testing are presented in table S3 in the supplements.

## Supplementary information

Supplementary Information 1.
