# Supplementary material for: Different functions of two putative Drosophila α2δ subunits in the same identified motoneurons
Source: Sci Rep. 2020 Aug 13;10:13670. doi: 10.1038/s41598-020-69748-8 (PMC7426832; doi:10.1038/s41598-020-69748-8)
Supplement: Supplementary file 1 — Supplementary Information 1. [file 41598_2020_69748_MOESM1_ESM.pdf]

Supplementary Information

Different functions of two putative Drosophila $\alpha_2\delta$ subunits in the same identified motoneurons

Laurin Heinrich and Stefanie Ryglewski*

Institute of Developmental Biology and Neurobiology,
Johannes Gutenberg University Mainz
Hanns-Dieter Hüsch Weg 15, 55128 Mainz
Germany

Corresponding author: *Stefanie Ryglewski at the above address.
Email: ryglewsk@uni-mainz.de

Table S1. Fly stocks used in this study

Genotype	use	identifier
y^{1} w^{67c23}; Mi{PT-GFSTF.2}CG4587$^{MI01722\text{-}GFSTF.2}$	CG4587 (stol) protein trap, endogenously expressing GFP fused to α_2 fragment of stol, resulting in stol^{GFP} [78]	RRID:BDSC_59289
y^{1} w*; Mi{PT-GFSTF.0}stj$^{MI00783\text{-}mCherry.0}$	stj protein trap, endogenously expressing mCherry fused to α_2 fragment of stj, resulting in stj^{mCherry}	this study
w^{1118}; P{KK106795}VIE-260B; P{$y^{+t7.7}$ $v^{+t1.8}$=TRiP.JF01825}attP2	*UAS-stol*^{RNAi} with *UAS-stj*^{RNAi} in the VALIUM10 vector [84]	RRID:FlyBase_FBst0479962; RRID:BDSC_25807
w^{1118}; P{KK101267}VIE-260B; P{w^{+mC}=UAS-Dcr-2.D}10	*UAS-stj*^{RNAi} with *UAS-dcr2* to enhance RNAi efficacy [52]	RRID:FlyBase_FBst0480379; RRID:BDSC_24651
w^{1118}; P{KK106795}VIE-260B; P{w^{+mC}=UAS-Dcr-2.D}10	*UAS-stol*^{RNAi} with *UAS-dcr2* to enhance RNAi efficacy [52]	RRID:FlyBase_FBst0479962; RRID:BDSC_24651
w^{1118}; P{$w^{+mW.hs}$=GawB}vGlut^{OK371}	*GAL4* expression under the control of *vGlut*^{OK371} (vesicular glutamate transporter). Expressed in all Drosophila motoneurons incl. larval crawling MNs.	RRID:BDSC_26160
w^{1118}; P{$y^{+t7.7}$ w^{+mC}=20XUAS-IVS-GCaMP6s}attP40; P{$y^{+t7.7}$ w^{+mC}=GMR23H06-GAL4}attP2	*UAS-GCaMP6s* is a genetically encoded Ca^{2+} indicator. *23H06-GAL4* from HHMI Janelia Farm GAL4 driver lines; associated gene *CG8084 anachronism*, *23H06-GAL4* expresses in pupal and adult DLM MNs and only very few others	RRID:BDSC_42746; 23H06-GAL4 discontinued at BDSC (available from our lab)
P{$w^{+mW.hs}$=GawB}elav^{C155}	*elav*^{C155}*-GAL4* is used for pan-neural expression of *UAS-RNAi* transgenes for Western Blot analysis and climbing assay	RRID:BDSC_458
w^{1118}; P{w^{+mC}=UAS-Dcr-2.D}2; P{$y^{+t7.7}$ w^{+mC}=GMR23H06-GAL4}attP2 P{w^{+mC}=UAS-myr-mRFP}2/TM6B, Tb^{1}	*UAS-dcr2* to enhance RNAi efficacy [39] along with *23H06-GAL4* with *UAS-myr-mRFP* on the same chromosome (Chr. 3) for easier identification of DLM MNs. for expression. Used for expression of double RNAi of stj and stol.	RRID:BDSC_24650; 23H06-GAL4 discontinued at BDSC (available from our lab)
w^{1118};; P{$y^{+t7.7}$ w^{+mC}=GMR23H06-GAL4}attP2, P{w^{+mC}=UAS-myr-mRFP}2/TM6B, Tb^{1}	*23H06-GAL4* along with *UAS-myr-mRFP* on the same chromosome (Chr. 3) for easier identification of DLM MNs.	23H06-GAL4 discontinued at BDSC (available from our lab); RRID:BDSC_7119
P{$w^{+mW.hs}$=GawB}elav^{C155}, cac$^{sfGFP\text{-}N}$	GFP tagged cacophony along with *elav*^{C155}*-GAL4* on the same chromosome. cacophony VGCCs are endogenously tagged with super folder (sf) GFP. GFP tag was inserted directly after the second translational start via CRISPR/cas9-based methods [53].	RRID:BDSC_458; identifier for cac$^{sf\ GFP\text{-}N}$ not yet assigned (40 Gratz et al. 2019)
w*; P{w^{+mC}=eve-GAL4.RN2}P, P{w^{+mC}=UAS-mCD8::GFP.L}LL5/CyO; P{w^{+mC}=Act(FRT.stop)GAL4}, P{w^{+mC}=UAS-FLP.D}JD2	inducible FLP out in larval MN1s and MN1b crawling MNs to induce mosaic expression of UAS-transgenes after second instar (*eve-GAL4* is turned off before third instar)	RRID:BDSC_7475; RRID:BDSC_4540; fly strain courtesy of Dr. S. Sanyal, Calico labs, San Francisco
w*;P{w^{+mC}=UAS-mCD8::GFP.L}LL5; P{$w^{+mW.hs}$=GawB}D42	GAL4-expression in motoneurons (incl. DLM MNs) under the control of the toll6 receptor	RRID:BDSC_8816
y^{1} M{vas-int.B}ZH-2A w*; sna^{Sco}/CyO, P{$ry^{+t7.2}$=sevRas1.V12}FK1	ϕC31 integrase expression under the control of *vasa*, which is active in the germ line	RRID:BDSC_36312
y^{1} w*; Mi{$y^{+mDint2}$=MIC}stj^{MI00783}/SM6a	fly strain carrying a MiMIC within a *stj* intron	RRID:BDSC_34109
y^{1} w*;CyO/sna^{Sco}	balancer strain	lab stock

Table S2. Genotypes used for experiments

Genotype	experiment	figure
y^{1} *w*; Mi{PT-GFSTF.0}stj*$^{MI00783-mCherry.0}$	stj^{mCherry}, used for Western Blot analysis of Drosophila $\alpha_2\delta$ subunits	Figs. 1A, B, E, 2A, Ai
y^{1} *w*^{67c23}*; Mi{PT-GFSTF.2}CG4587*$^{MI01722-GFSTF.2}$	stol^{GFP}, used for Western Blot analysis of Drosophila $\alpha_2\delta$ subunits	Figs. 1A, B, D, 2A, Ai
**pan-neural *stol*$^{GFP\ RNAi}$ for RNAi efficacy with Western:**<br>*P{w*$^{+mW.hs}$*=GawB}elav*^{C155}*; Mi{PT-GFSTF.2}CG4587*$^{MI01722-GFSTF.2}$ */ P{KK106795}VIE-260B; P{w*$^{+mC}$*=UAS-Dcr-2.D}10 / +*	*stol*^{RNAi} under the control of the pan-neural driver *elav*^{C155}*-GAL4* along with UAS-Dcr-2 to enhance RNAi efficacy in *stol*$^{GFP\ RNAi}$ background used in Western Blot analysis of RNAi efficacy.	Figs. 1B, Bi
**control for *stol*$^{GFP\ RNAi}$ for RNAi efficacy with Western:**<br>*P{w*$^{+mW.hs}$*=GawB}elav*^{C155}*; Mi{PT-GFSTF.2}CG4587*$^{MI01722-GFSTF.2}$ */ +; P{w*$^{+mC}$*=UAS-Dcr-2.D}10 / +*	Pan-neural expression of UAS-Dcr-2 under the control of *elav*^{C155}*-GAL4* in animals with endogenous expression of stol^{GFP} as control for RNAi efficacy via Western Blot. Used as control for *stol*^{RNAi} in stol^{GFP} animals in Western Blot.	Figs. 1B, Bi
**pan-neural *stj*$^{mCherry\ RNAi}$ for RNAi efficacy with Western:**<br>*P{w*$^{+mW.hs}$*=GawB}elav*^{C155}*; Mi{PT-GFSTF.0}stj*$^{MI00783-mCherry.0}$*/ P{KK101267}VIE-260B; P{w*$^{+mC}$*=UAS-Dcr-2.D}10/+*	*stj*^{RNAi} under the control of the pan-neural driver *elav*^{C155}*-GAL4* along with *UAS-Dcr-2* to enhance RNAi efficacy in stj^{mCherry} background, used in Western Blot analysis of RNAi efficacy	Figs. 1B, Bi
**control for *stj*$^{mCherry\ RNAi}$ for RNAi efficacy with Western:**<br>*P{w*$^{+mW.hs}$*=GawB}elav*^{C155}*; Mi{PT-GFSTF.0}stj*$^{MI00783-mCherry.0}$*/ +; P{w*$^{+mC}$*=UAS-Dcr-2.D}10/+*	Pan-neural expression of *UAS-Dcr-2* under the control of *elav*^{C155}*-GAL4* in animals with endogenous expression of stj^{mCherry} as control for RNAi efficacy via Western Blot	Figs. 1B, Bi
**pan-neural *stol*^{RNAi} in climbing assay:**<br>*P{w*$^{+mW.hs}$*=GawB}elav*^{C155}*; P{KK106795}VIE-260B /P{w*$^{+mC}$*=UAS-Dcr-2.D}2*	pan-neural *stol*^{RNAi} under the control of *elav*^{C155}*-GAL4* with co-expression of *UAS-Dcr-2* to enhance RNAi efficacy for testing of stol neuronal function in climbing assay.	Fig. 1C
**control for climbing assay:**<br>*P{w*$^{+mW.hs}$*=GawB}elav*^{C155}*; P{w*$^{+mC}$*=UAS-Dcr-2.D}2 /+*	control for climbing assay	Fig. 1C
**test of compensation of stol^{GFP} in pan-neural *stj*^{RNAi}:**<br>*P{w*$^{+mW.hs}$*=GawB}elav*^{C155}*; Mi{PT-GFSTF.2}CG4587*$^{MI01722-GFSTF.2}$ */ P{KK101267}VIE-260B; P{w*$^{+mC}$*=UAS-Dcr-2.D}10 / +*	*stj*^{RNAi} in stol^{GFP} background to test whether stol may compensate for the absence stj	Fig. 1D
**test of compensation of stj^{mCherry} in pan-neural *stol*^{RNAi}:**<br>*P{w*$^{+mW.hs}$*=GawB}elav*^{C155}*; Mi{PT-GFSTF.0}stj*$^{MI00783-mCherry.0}$ */ P{KK106795}VIE-260B; P{w*$^{+mC}$*=UAS-Dcr-2.D}10 /+*	*stol*^{RNAi} in stj^{mCherry} background to test whether stj may compensate for the absence of stol	Fig. 1E
**synaptic transmission experiments with *stj*^{RNAi} in larval MNs:**<br>*w*^{1118}*; P{w*$^{+mW.hs}$*=GawB}vGlut*^{OK371}*/ P{KK101267}VIE-260B; P{w*$^{+mC}$*=UAS-Dcr-2.D}10/+*	*stj*^{RNAi} under the control of *vGlut*^{OK371}*-GAL4* (expression in MNs, including larval crawling MNs), used for larval muscle recordings (EPSPs)	Figs. 2A, Ai
**synaptic transmission experiments with *stol*^{RNAi} in larval MNs:**<br>*w*^{1118}*; P{w*$^{+mW.hs}$*=GawB}vGlut*^{OK371}*/ P{KK106795}VIE-260B; P{w*$^{+mC}$*=UAS-Dcr-2.D}10/+*	*stol*^{RNAi} under the control of *vGlut*^{OK371}*-GAL4* (expression in MNs, including larval crawling MNs), used for larval muscle recordings (EPSPs)	Figs. 2A, Ai
**control for larval synaptic transmission experiments:**<br>*w*^{1118}*; P{w*$^{+mW.hs}$*=GawB}vGlut*^{OK371}*/+; P{w*$^{+mC}$*=UAS-Dcr-2.D}10/+*	expression of *UAS-Dcr-2* in all MNs incl. larval crawling MNs MN1s and MN1b, control for *stj*^{RNAi} and *stol*^{RNAi} in larval muscle recordings (EPSPs)	Figs. 2A, Ai
***stj*^{RNAi} in third instar larval MN1s and MN1b crawling MNs for Ca^{2+} current recordings:**<br>*w*; P{w*$^{+mC}$*=eve-GAL4.RN2}P, P{w*$^{+mC}$*=UAS-mCD8::GFP.L}LL5/ P{KK101267}VIE-260B; P{w*$^{+mC}$*=Act(FRT.stop)GAL4}, P{w*$^{+mC}$*=UAS-FLP.D}JD2/ P{w*$^{+mC}$*=UAS-Dcr-2.D}10/+*	Mosaic expression of *stj*^{RNAi} under the control of the even skipped (eve) promoter (expression in larval MN1s and MN1b crawling MNs). *eve* is turned off before third instar (which was used here). This was prevented by driving UAS-FLP under the control of *eve-GAL4*, which cut the FRT flanked stop between *actin* and *GAL4* so that *UAS-transgene* expression continued under the control of the very strong actin-promoter. *UAS-mCD8::GFP* was co-expressed along with *UAS-Dcr-2* to enhance RNAi efficacy. UAS-transgene expression occurred in a mosaic fashion, reported by GFP. This stock was used for all larval Ca^{2+} current recordings expressing *stj*^{RNAi} because it allows recording internal controls.	Figs. 2B-C
***stol*^{RNAi} in third instar larval MN1s and MN1b crawling MNs for Ca^{2+} current recordings:**<br>*w*; P{w*$^{+mC}$*=eve-GAL4.RN2}P, P{w*$^{+mC}$*=UAS-mCD8::GFP.L}LL5/ P{KK106795}VIE-260B; P{w*$^{+mC}$*=Act(FRT.stop)GAL4}, P{w*$^{+mC}$*=UAS-FLP.D}JD2/ P{w*$^{+mC}$*=UAS-Dcr-2.D}10/+*	Mosaic expression of *stol*^{RNAi} under the control of the even skipped (eve) promoter (expression in larval MN1s and MN1b crawling MNs). *eve* is turned off before third instar (which was used here). This was prevented by driving *UAS-FLP* under the control of *eve-GAL4* which cut the FRT flanked stop between actin and GAL4 so that *UAS-transgene* expression continued under the control of the very strong actin-promoter. *UAS-mCD8::GFP*	Figs. 2B-C

	was co-expressed along with *UAS-Dcr-2* to enhance RNAi efficacy. UAS-transgene expression occurred in a mosaic fashion, reported by GFP. This stock was used for all larval Ca^{2+} current recordings expressing *stol*^{RNAi} because it allows recording internal controls.	
**control for *stj*^{RNAi} and *stol*^{RNAi} in third instar larval MN1s and MN1b crawling MNs for Ca^{2+} current recordings:** *w*; P{w^{+mC}=eve-GAL4.RN2}P, P{w^{+mC}=UAS-mCD8::GFP.L}LL5/+; P{w^{+mC}=Act(FRT.stop)GAL4}, P{w^{+mC}=UAS-FLP.D}JD2/ P{w^{+mC}=UAS-Dcr-2.D}10/+*	Mosaic expression of *UAS-Dcr-2* under the control of the even skipped (*eve*) promoter (expression in larval MN1s and MN1b crawling MNs). *eve* is turned off before third instar (which was used here). This was prevented by driving UAS-FLP under the control of *eve-GAL4,* which cut the FRT flanked stop between actin and GAL4 so that UAS-transgene expression continued under the control of the very strong actin-promoter. *UAS-mCD8::GFP* was co-expressed UAS-transgene expression occurred in a mosaic fashion, reported by GFP. This stock was used as control for all larval Ca^{2+} current recordings expressing *stj*^{RNAi} or *stol*^{RNAi}	Figs. 2B-C
***stj*^{RNAi} in adult and pupal DLM MNs for Ca^{2+} current recordings:** *w^{1118}; P{KK101267}VIE-260B/+; P{y$^{+t7.7}$ w^{+mC}=GMR23H06-GAL4}attP2, P{w^{+mC}=UAS-myr-mRFP}2/ P{w^{+mC}=UAS-Dcr-2.D}10*	*stj*^{RNAi} under the control of *23H06-GAL4* (expression in DLM MNs) with *UAS-myr-mRFP* which results in punctate red MN label for better identification. We could not detect any negative impact on Ca^{2+} currents or action potentials when *UAS-myr-mRFP* is expressed in DLM MNs. Co-expression of UAS-Dcr-2 for higher RNAi efficacy. Used for adult and pupal Ca^{2+} current and pupal action potential recordings.	Figs. 2D-G (adult), Figs. 2H-I (pupae)
***stol*^{RNAi} in adult and pupal DLM MNs for Ca^{2+} current recordings:** *w^{1118}; P{KK106795}VIE-260B/+; P{y$^{+t7.7}$ w^{+mC} =GMR23H06-GAL4}attP2, P{w^{+mC}=UAS-myr-mRFP}2/ P{w^{+mC}]=UAS-Dcr-2.D}10*	*stol*^{RNAi} under the control of *23H06-GAL4* (expression in DLM MNs) with *UAS-myr-mRFP* which results in punctate red MN label for better identification. We could not detect any negative impact on Ca^{2+} currents or action potentials when *UAS-myr-mRFP* is expressed in DLM MNs. Co-expression of *UAS-Dcr-2* for higher RNAi efficacy. Used for adult and pupal Ca^{2+} current and pupal action potential recordings.	Figs. 2D-G (adult), Figs. 2H-I (pupae)
***stj*^{RNAi} and *stol*^{RNAi} (double RNAi) in pupal DLM MNs for Ca^{2+} current recordings:** *w^{1118}; P{KK106795}VIE-260B /P{w^{+mC}=UAS-Dcr-2.D}10; P{ y$^{+t7.7}$ w^{+mC}=GMR23H06-GAL4}attP2, P{ w^{+mC}=UAS-myr-mRFP}2/ P{y$^{+t7.7}$ v$^{+t1.8}$=TRiP.JF01825}attP2*	*stj*^{RNAi} and *stol*^{RNAi} under the control of *23H06-GAL4* (expression in DLM MNs) with *UAS-myr-mRFP* which results in punctate red MN label for better identification. We could not detect any negative impact on Ca^{2+} currents or action potentials when *UAS-myr-mRFP* is expressed in DLM MNs. Co-expression of *UAS-Dcr-2* for higher RNAi efficacy. Used for adult and pupal Ca^{2+} current and pupal action potential recordings.	Figs. 2H-I
**control for *stj*^{RNAi} and *stol*^{RNAi} in adult and pupal DLM MNs for Ca^{2+} current recordings:** *w^{1118};; P{y$^{+t7.7}$ w^{+mC}=GMR23H06-GAL4}attP2, P{w^{+mC}=UAS-myr-mRFP}2/ P{w^{+mC}=UAS-Dcr-2.D}10*	expression of *UAS-Dcr-2* under the control of *23H06-GAL4* as control for single and double *UAS-stj*^{RNAi} and *UAS-stol*^{RNAi}	Figs. 2D-G (adult), Figs. 2H-I (pupae)
***stol*^{RNAi} and cac^{GFP} for axonal label:** *P{w$^{+mW.hs}$=GawB}elav^{C155}, cac$^{sfGFP-N}$; P{KK106795}VIE-260B/+; P{w^{+mC}=UAS-Dcr-2.D}10/+*	pan-neurally expressed *stol*^{RNAi} (under the control of *elav^{C155}-GAL4*) along with endogenously GFP-tagged cacophony VGCC channels for visualization in the axon	Figs. 3A, B
***stj*^{RNAi} and cac^{GFP} for axonal label:** *P{w$^{+mW.hs}$=GawB}elav^{C155}, cac$^{sfGFP-N}$; P{KK101267}VIE-260B /+; P{w^{+mC}=UAS-myr-mRFP}2/ P{w^{+mC}=UAS-Dcr-2.D}10*	pan-neurally expressed *stj*^{RNAi} (under the control of *elav^{C155}-GAL4*) along with endogenously GFP-tagged cacophony VGCC channels for visualization in the axon	Figs. 3A, B
**control for for axonal cac^{GFP} label:** *P{w$^{+mW.hs}$=GawB}elav^{C155}, cac$^{sfGFP-N}$; +/+; P{w^{+mC}=UAS-Dcr-2.D}10/+*	control visualization of cacophony^{GFP} VGCCs in the axon	Figs. 3A, B
***stj*^{RNAi} in pupal DLM MNs for pupal AP recordings and adult intracellular DLM MN fill:** *w*;P{KK101267}VIE-260B/ P{w^{+mC}=UAS-mCD8::GFP.L}LL5; P{w$^{+mW.hs}$=GawB}D42/ P{w^{+mC}=UAS-Dcr-2.D}10*	*stj*^{RNAi} under the control of D42-GAL4 (incl. expression in DLM MNs). Co-expression of *UAS-Dcr-2* for higher RNAi efficacy. Used for pupal action potential recordings.	Figs. 3C, D; Fig. 5
***stol*^{RNAi} in pupal DLM MNs for pupal AP recordings and adult intracellular DLM MN fill:** *w*;P{KK106795}VIE-260B/ P{w^{+mC}=UAS-mCD8::GFP.L}LL5; P{w$^{+mW.hs}$=GawB}D42/ P{w^{+mC}=UAS-Dcr-2.D}10*	*stol*^{RNAi} under the control of *D42-GAL4* (incl. expression in DLM MNs). Co-expression of *UAS-Dcr-2* for higher RNAi efficacy. Used for pupal action potential recordings.	Figs. 3C, D; Fig. 5
**control for *stj*^{RNAi} and *stol*^{RNAi} for pupal AP recordings and adult intracellular DLM MN fill:**	control, expresses *UAS-Dcr-2* under the control of *D42-GAL4*	Figs. 3C, D; Fig. 5

w;P{w^{+mC}=UAS-mCD8::GFP.L}LL5 /+; P{$w^{+mW.hs}$=GawB}D42/ P{w^{+mC}=UAS-Dcr-2.D}10*		
**pupal Ca^{2+} imaging upon induced AP firing in *stj^{RNAi}*:** *w^{1118}; P{ $y^{+t7.7}$ w^{+mC}=20XUAS-IVS-GCaMP6s}attP40/ P{KK101267}VIE-260B; P{ $y^{+t7.7}$ w^{+mC}=GMR23H06-GAL4}attP2/ P{w^{+mC} =UAS-Dcr-2.D}10*	*stj^{RNAi}* under the control of *23H06-GAL4* (expression in DLM MNs) with the genetically encoded green Ca^{2+} indicator UAS-GCaMP6s Co-expression of *UAS-Dcr-2* for higher RNAi efficacy. Used for pupal Ca^{2+} imaging experiments.	Fig. 4
**pupal Ca^{2+} imaging upon induced AP firing in *$stol^{RNAi}$*:** *w^{1118}; P{ $y^{+t7.7}$ w^{+mC}=20XUAS-IVS-GCaMP6s}attP40/ P{KK101267}VIE-260B; P{ $y^{+t7.7}$ w^{+mC}=GMR23H06-GAL4}attP2/ P{ w^{+mC}=UAS-Dcr-2.D}10*	*$stol^{RNAi}$* under the control of *23H06-GAL4* (expression in DLM MNs) with the genetically encoded green Ca^{2+} indicator UAS-GCaMP6s Co-expression of *UAS-Dcr-2* for higher RNAi efficacy. Used for pupal Ca^{2+} imaging experiments.	Fig. 4
**control for pupal Ca^{2+} imaging upon induced AP firing:** *w^{1118}; P{$y^{+t7.7}$ w^{+mC}=20XUAS-IVS-GCaMP6s}attP40/+; P{$y^{+t7.7}$ w^{+mC}=GMR23H06-GAL4}attP2/ P{ w^{+mC}=UAS-Dcr-2.D}10*	control, expresses *UAS-GCaMP6s* and *UAS-Dcr-2* under the control of *23H06-GAL4*	Fig. 4
y^1 M{vas-int.B}ZH-2A w; sna^{Sco}/ Mi{$y^{+mDint2}$=MIC}$stj^{MI00783}$*	stage two embryos used for injection of mCherry plasmid for generation of $stj^{mCherry}$ protein trap strain	

Table S3: Statistical test results

Experiment (Figure)	Test	F-value	p-value of test	p-value ctrl vs. *stj*^{RNAi}	p-value ctrl vs. *stol*^{RNAi}	p-value ctrl vs. double RNAi	p-value *stj*^{RNAi} vs. *stol*^{RNAi}	p-value *stj*^{RNAi} vs. double RNAi	p-value *stol*^{RNAi} vs. double RNAi
**Climbing speed (1C)**	Student's T-test, unpaired				$1,7*10^{-12}$				
**Western Blot stol in *stj*^{RNAi} (1D)**	Mann-Whitney U-test			0.81					
**Western Blot stj in *stol*^{RNAi} (1E)**	Mann-Whitney U-test				0.63				
**EPSP amplitude larva (2A, Ai)**	one way ANOVA with LSD post-hoc	F(2,39) = 12.723	$5.6*10^{-5}$	$2.77*10^{-4}$	0.282		$2.2*10^{-5}$		
**Ca_v2^{GFP} abundance at larval NMJ (2B, Bi)**	one way ANOVA with Games-Howell post-hoc	F(2,23) = 63,952	$4*10^{-10}$	$1.6*10^{-5}$	0.21		$8*10^{-6}$		
**larval Ca^{2+} current (2C, D)**	one way ANOVA with LSD post-hoc	F(2,32) = 13.586	$5.4*10^{-4}$	$1.45*10^{-4}$	0.367		$1.02*10^{-4}$		
**adult sustained Ca^{2+} current (2E, G)**	one way ANOVA with LSD post-hoc	F(2,11) = 9,781	0.004	0.006	0.398		0.002		
**adult transient Ca^{2+} current (2F, H)**	one way ANOVA with LSD post-hoc	F(2,11) = 5.881	0.018	0.025	0.456		0.008		
**pupal Ca^{2+} current (2I, J)**	one way ANOVA with Tukey post-hoc	F(3,33) = 13.538	$6*10^{-6}$	$8.6*10^{-5}$	0.921	0.012	$8.3*10^{-5}$	0.851	0.006
**axonal Ca_v2^{GFP} abundance (3A, B)**	one way ANOVA with LSD post-hoc	F(2,25) = 23.253	$2*10^{-6}$	0.006	0.001		$3.98*10^{-7}$		
**% change of AP width after Cd^{2+} (3C, D)**	Median test for k independent samples		$6.16*10^{-8}$	$8.7*10^{-5}$	0.016	$6*10^{-8}$	$5.2*10^{-5}$	0.018	$3.1*10^{-5}$
**% change of Ca^{2+} signal in dendrites (4B)**	Kruskal Wallis ANOVA with Dunn post hoc		$2.61*10^{-6}$	0.001	$4*10^{-4}$	$1,06*10^{-7}$	0.674	0.274	0.282
**% change of Ca^{2+} signal in axons (4Bi)**	Kruskal Wallis ANOVA with Dunn post hoc		$1.36*10^{-11}$	$1.53*10^{-7}$	0.031	0.003	$3.41*10^{-11}$	0.03	$4*10^{-6}$
**total dendritic length (5D)**	one way ANOVA with Games-Howell post-hoc	F(2,12) = 14.499	0.001	$2.37*10^{-4}$	0.009		0.794		
**# dendritic branches (5E)**	one way ANOVA with LSD post-hoc	F(2,12) = 7.354	0.008	0.005	0.007		0.608		
**mean dendrite length (5F)**	one way ANOVA with Games-Howell post-hoc	F(2,12) = 0.531	0.601						
**mean path length (5G)**	Kruskal Wallis ANOVA with Dunn post hoc		0.415						

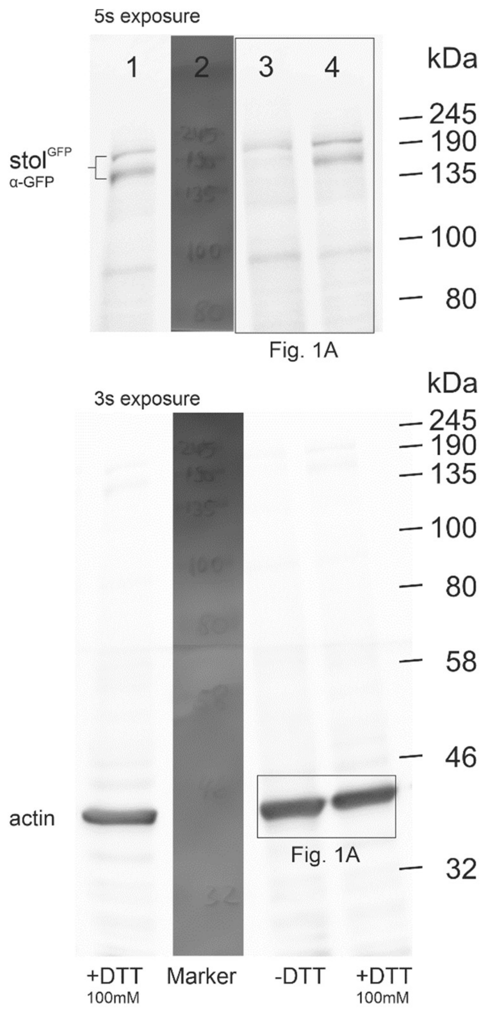

Figure S1, Heinrich, Ryglewski 2020

Western Blot with and without reducing agent DTT

Figure S1: $\alpha_2\delta$ is cleaved into two parts by the reducing agent dithiotreitol (DTT).

Original Western Blot of L3 larval ventral nerve cords (VNCs, 20 per lane) of stol^{GFP} larvae (*yw;stol^{GFP};+*) with and without the reducing agent DTT (relates to figure 1A). The GFP tag used for detection due to lack of specific Drosophila $\alpha_2\delta$ antibodies resides close to the N-terminus of the α_2-moiety. Exposure time was 5s for detection of stol^{GFP} (top), and 3s for the actin loading control (bottom). 3s exposure time resulted in low intensity stol^{GFP} bands (bottom, faint bands in upper part of the blot). The presence of 100 mM DTT resulted in two bands that were ~25 kDa apart (lanes 1 and 4), which corresponds to the predicted molecular weight of the δ moiety. Absence of DTT resulted in only the larger of the two bands (lane 3), likely representing un-cleaved $\alpha_2\delta$. From this we conclude that in the presence of 100 mM DTT a portion of $\alpha_2\delta$ is cleaved into α_2 and δ resulting in two bands, both carrying the tag. δ alone is not detectable because it does not carry a tag. Contrast of the entire image was enhanced. The gel was slightly tilted which was not corrected during image production. A pre-stained molecular weight marker (25 – 245 kDa) was used (lane 2). Lanes 3 and 4 were used for figure 1A. Intensity in the top image was adjusted linearly.

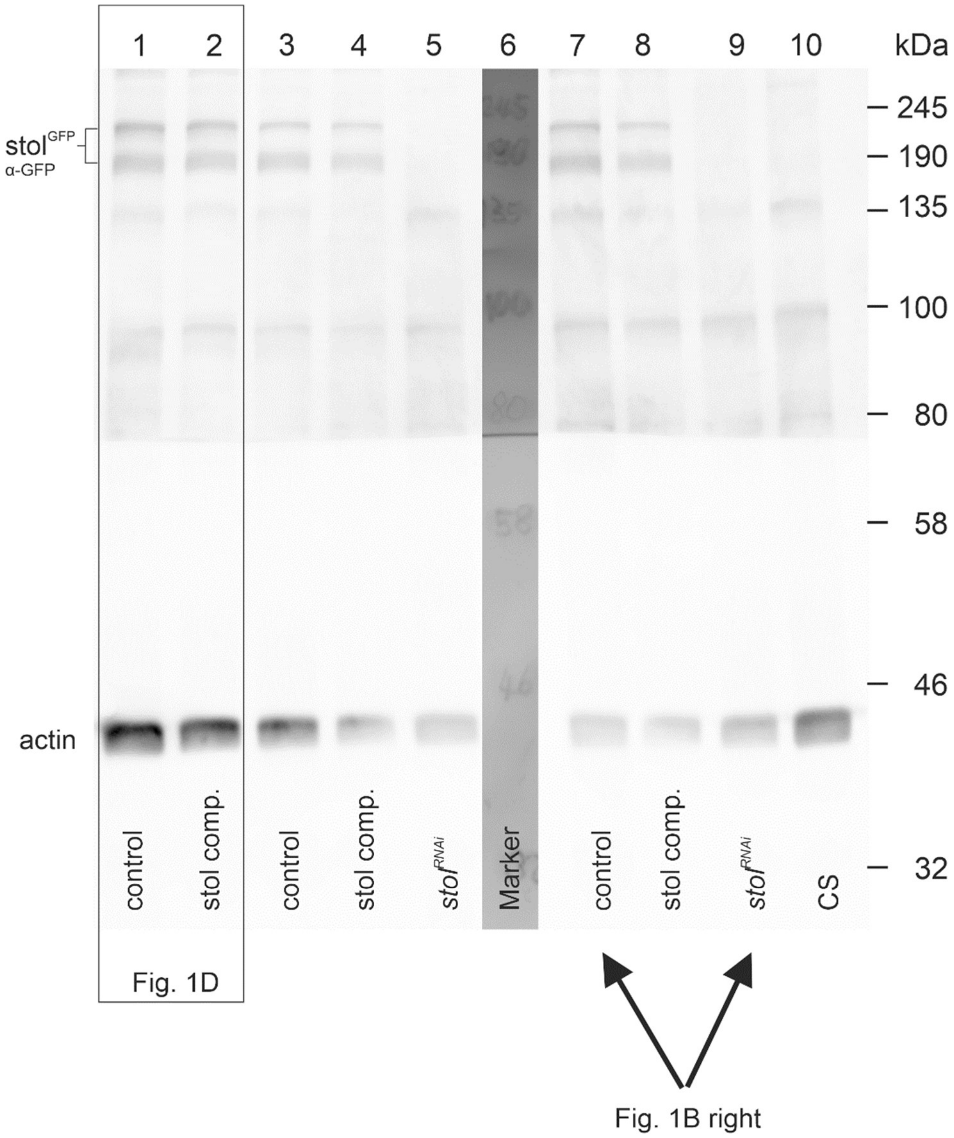

Figure S2, Heinrich, Ryglewski 2020

Western Blot for *stol*^{RNAi}
and stol compensation following *stj*^{RNAi}

Figure S2: Western Blot validation of *stol*^{RNAi} and compensation assay of stol following *stj*^{RNAi}.

Original Western Blot for the analysis of *stol*^{RNAi} efficacy (relates to figure 1B, right) and possible compensation of stol following *stj*^{RNAi} (relates to figure 1D). Pan-neural *stol*^{RNAi} was driven with *elav*^{C155}*-GAL4* and knock down efficacy was enhanced by co-expression of *UAS-dcr2* (Dietzl et al., 2007; genotype: *elav*^{C155}*-GAL4>stol*^{GFP}*/UAS-stol*^{RNAi}*;UAS-dcr2*, lanes 5 and 9). Stol was detected via endogenously expressed GFP tag using α-GFP antibody. Possible compensation of stol following pan-neural *stj*^{RNAi} was assessed (stol comp.: *elav-*^{C155}*-GAL4>UAS-stj*^{RNAi}*/stol*^{GFP}*;UAS-dcr2*, lanes 2, 4, and 8). As positive control *elav*^{C155}*-GAL4>stol*^{GFP}*;UAS-dcr2* (lanes 1, 3, and 7) and as negative control for un-tagged $\alpha_2\delta$ Canton Special (CS) wildtype (lane 10) were used. Loading control was actin at 45 kDa. 20 VNCs per lane were used. A pre-stained molecular weight marker (25 – 245 kDa) was used (lane 6). Exposure time was 8s.

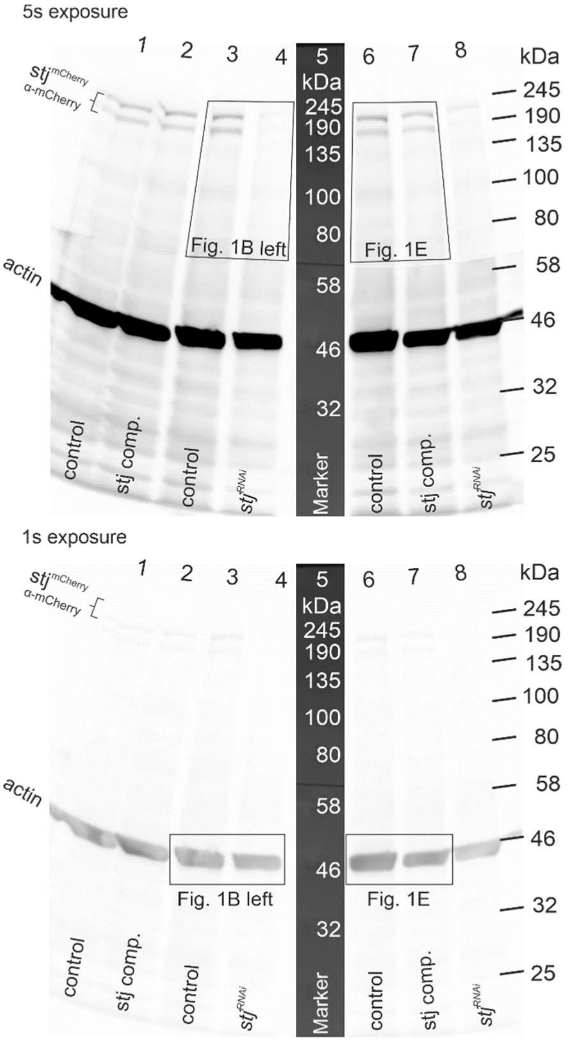

Figure S3, Heinrich, Ryglewski 2020

Western Blot for *stj*^{RNAi}
and stj compensation following *stol*^{RNAi}

Figure S3: Western Blot validation of *stj*^{RNAi} and compensation assay of stj following *stol*^{RNAi}.

Original Western Blot for the analysis of *stj*^{RNAi} efficacy (relates to figure 1B, left) and possible compensation of stj following *stol*^{RNAi} (relates to figure 1E). Bands were detected at two different exposure times, 5s (top) and 1s (bottom), due to over-exposure of the actin loading control. Figures 1B left and 1E were created from the top image for *stj*^{RNAi} and stj compensation (stj comp.) with their respective loading controls taken from the bottom image. Pan-neural *stj*^{RNAi} was driven with *elav*^{C155}-*GAL4* and knock down efficacy was enhanced by co-expression of *UAS-dcr2* (Dietzl et al., 2007; genotype: *elav*^{C155}*-GAL4>stj*^{mCherry}*/UAS-st*^{RNAi}*;UAS-dcr2*, lanes 4 and 8). Stj was detected via endogenously expressed mCherry tag using α-mCherry antibody. Possible compensation of stj following pan-neural *stol*^{RNAi} was assessed (stj comp.: *elav-*^{C155}*-GAL4>UAS-stol*^{RNAi}*/stj*^{mCherry}*;UAS-dcr2*, lanes 2 and 7). As positive control *elav*^{C155}*-GAL4>stj*^{mCherry}*;UAS-dcr2* (lanes 1, 3 and 6) was used. Loading control was actin at 45 kDa. 20 VNCs per lane were used. A pre-stained molecular weight marker (25 – 245 kDa) was used (lane 5). The running front of the gel was not even resulting in a semi-circular appearance.
